# Supplementary material for: The rate and potential relevance of new mutations in a colonizing plant lineage
Source: PLoS Genet. 2018 Feb 12;14(2):e1007155. doi: 10.1371/journal.pgen.1007155 (PMC5825158; doi:10.1371/journal.pgen.1007155)
Supplement: S1 Text — (PDF) [file pgen.1007155.s001.pdf]

Text S1: Detailed methods and analyses

1. Sample collection and preparation 1
2. Authenticity of aDNA 1
3. SNP calling thresholds 1
4. Resequencing of Col-0 Mutation Accumulation lines 1
5. Identification of bona fide HPG1 accessions and mutations 2
    5.1 HPG1 and other haplogroups in North America 2
    5.2 North american private diversity 2
6. Extent of linkage disequilibrium and recombination 3
7. Substitution and mutation rate analyses 3
    7.1 Greenhouse grown MA lines 3
    7.2 Natural populations of HPG1 4
        7.2.1 Net distances 4
        7.2.2 Bayesian tip-calibration 4
        7.2.3 Methylation status of mutated sites 5
8. Phenotypic association analyses and dating of newly arisen mutations 6
    8.1. Phenotyping 6
        8.1.1 Root 6
        8.1.2 Seed size 6
        8.1.3 Flowering in the growth chamber 6
        8.1.4 Fecundity in the field 7
    8.2 Quantitative genetic analyses 7
        8.2.1 Heritability 7
        8.2.2 Linear Models 8
        8.2.3 Evaluation of significance 8
        8.2.4 Context of de novo mutations associated with phenotypes 9
        8.2.5 Functional information 9
        8.2.6 Proof of concept examples 9

References **11**

1. Sample collection and preparation

Seeds from modern accessions (Table S1) were bulked at the University of Chicago. Progeny for DNA extraction was grown at the Max Planck Institute for Developmental Biology. We used 2 to 8 mm^2 of dried tissue for destructive sampling from the herbarium specimens (Table S1).

2. Authenticity of aDNA

First, unrepaired sequencing herbarium libraries were screened for authenticity by sequencing at low coverage on Illumina HiSeq 2500 or MiSeq instruments. To verify the DNA retrieved from historical samples of *A. thaliana* was authentic, we checked the percentage of endogenous DNA of the sample (Fig. S1A) as well as typical postmortem DNA damages: high fragmentation of DNA (Fig. S1B), enrichment of substitution from C to T at the first base pair (Fig. S1C) as well as purine enrichment at breakpoints of DNA fragments (Fig. S1D) (for details see [74]). Sequencing to produce the final genomes (101 bp paired end) was carried out on an Illumina HiSeq 2000 instrument after DNA repair by uracil-DNA glycosylase [53,54,75]. For a detailed analysis of authenticity in a fraction of our samples, see Weiss et al. [74].

3. SNP calling thresholds

To assess the effect of SNP calling thresholds on the mutation rate, we employed three different SHORE v0.9.0 quality thresholds following previous work (see Table S4 from [76]): allowing at most one intermediate penalty in all strains (most stringent threshold; "32-32"); requesting that at least one strain had at most one intermediate penalty, while all others were allowed up to two high and one intermediate penalties (intermediate stringency, "32-15"); and finally allowing one high and one intermediate penalty for all strains (most lenient stringency, "24-24"). On top of that, we would either allow missing information per SNP in up to 50% of accessions, or request complete information (0% missing rate). Thus, the most rigorous case would be 32-32 quality and 0% missing rate, and the most relaxed 24-24 quality and 50% maximum missing rate. Substitution rate calculations (section 7.2) were done for datasets from all combinations of these quality parameters (Fig. S3), and we chose the regular 32_15 quality threshold and complete information for the final estimate (Fig 3 C, E).

4. Resequencing of Col-0 Mutation Accumulation lines

We also sequenced the genomes of twelve greenhouse-grown mutation accumulation (MA) lines, including ten that had been sequenced at lower coverage before [76,77] (Table S2). We called SNPs, indels and structural variants (SVs), following the workflow and parameters described [78], but

without iterations. This procedure resulted in 2,203 polymorphisms shared by all lines, indicating errors in the reference sequence (12% of variants replaced N's in the TAIR9 genome) or genetic differences in the founder plant of the MA population compared to the Col-0 reference genome. In addition, we identified 388 segregating variants across the twelve lines (Table S2), of which 350 were singletons. This analysis revealed on average 25.5 SNPs, 4.9 deletions and 3.2 insertions per MA line at the 31st generation (Table S2), compared to 19.6 SNPs, 2.4 deletions and 1.0 insertions previously detected in the 30th generation with shorter read length and lower read depth [79]. The genome length accessed in this sequencing effort, 115,954,227 bp, was used to scale the number of point mutations to a rate of 7.1×10^{-9} mutations $site^{-1}$ $generation^{-1}$ (Table S3, Fig. 3E).

5. Identification of *bona fide* HPG1 accessions and mutations

5.1 HPG1 and other haplogroups in North America

The modern samples had been originally selected based on previous genotyping efforts of about 2,000 N. American accessions with for 149 nuclear, intermediate-frequency SNPs. This work had pointed to there being a single haplogroup, HPG1, that was invariant at these 149 markers and that accounted for about half of N. American individuals genotyped [80]. We extracted from the 123 genomes we had completely sequenced the same 149 SNPs and built a neighbour joining tree (Fig. S1A). We also built the same tree with the whole-genome sequences (Fig. S1B), which was mostly in agreement with the 149 SNP tree.

The previous work had identified several other haplogroup in N. America [80]. Not surprisingly, HPG1 individuals outcross with other lineages, and this accounts for some of the individuals which we later removed, because they did not agree completely in all 149 markers with the HPG1 consensus.

5.2 North american private diversity

Having identified these *bona fide* HPG1 individuals, we wanted to confirm that the diversity has a legitimate origin from *de novo* mutations. For that we used the 1001 Genomes resource (www.1001genomes.org), which covers a sampling of populations from the native Eurasian and African range. Subsetting the genomes from this resource to only European accessions, and limiting the SNP set to those with ≥1% frequency of alternative alleles and a maximum of 50% missing data (the same quality rate as our HPG1 SNP call), there were 300 variants out of all 5,181 HPG1 variants that were also found in Europe or Asia (5.7%). Changing the maximum missing data to 10% we get a more conservative estimate of 1.8% overlap, while increasing the maximum missing data to 90%, we get the anti-conservative estimate of 6.5% overlap. Only one of the reported SNPs associated with phenotypes (see Section 8) was among these shared variants.

There are several scenarios that can explain these shared SNPs. One is simply that there was not a single founding seed, but a few of closely related individuals coming from the native range. Other explanations are that parallel mutations occurred in North America and Eurasia, that HPG1 individuals were reintroduced to Europe, or that reversion-mutation occurred in some HPG1 individuals. The latter is not implausible given the large population size of the species and the fact that about 10% of all sites in the genome are SNPs in the 1001 Genomes collection. As explained in the main text, SNP sharing due to admixture with other lineages is extremely unlikely, as such cases should be evident as blocks of high SNP diversity along the genome (Fig. 4).

Finally, regarding chloroplast diversity, we did not find any SNP in the chloroplast of HPG1 individuals. This is probably because chloroplast mutation rates are much slower [81] and because the founder colonizers actually came from a small batch of seeds from an identical mother (chloroplast diversity in the native range is of 2,842 SNPs [82]).

6. Extent of linkage disequilibrium and recombination

We estimated pairwise linkage disequilibrium (LD) between all possible combinations of informative sites, ignoring singletons, by computing r^2, D and D' statistics. LD decay was estimated using a linear regression approach. Linkage disequilibrium parameter $|D'|$ did not decay with physical distance (intercept = 0.99, slope = 0.00) among all SNP pairs. Indeed 99.975% of pairwise SNP comparisons had $|D'|=1$ meaning that 99.975% of those comparisons only three out of the four possible gametes (ab, aB, Ab, AB) are found and thus mutation alone can explain their existence without the need of invoking recombination. In other words, such three gametes can be represented in a tree structure. LD and recombination related statistics were determined using DnaSP v5 [83].

7. Substitution and mutation rate analyses

7.1 Greenhouse grown MA lines

Mutation rates were estimated for each 31st generation greenhouse-grown MA line [76] as the number of mutations divided by the total bp length of the genome (or a given annotation) and by 31 generations (the two MA lines with only three generations were excluded from this analysis). Mean and confidence intervals across lines are reported (Table S3). The genome length was determined as all base pairs with coverage higher or equal to 3, and a SHORE mapping quality score of at least 32 in one sample (Table S2).

7.2 Natural populations of HPG1

7.2.1 Net distances

For the "net genetic distances" method, we computed confidence intervals of the *b* regression slope coefficient ($D' = a+bT'$) using a bootstrap with replacement of 1,000 samples to avoid over-confident confidence intervals due to lack of independence of points [30]. We used either all SNPs or SNPs at specific annotations to calculate different substitution rates and scaled the slope into a per-base rate using all positions (of the given annotation) that passed alternative or reference call quality thresholds rather than using a single value of genome length (Table S3). For all annotations we calculated substitution rates with three quality thresholds and either full information per SNP or allowing a maximum of 50% missing accessions per SNP (see Section 3 and Fig. S1C).

For some annotations substitution rates were not reliable. For instance, in 3' and 5' UTR regions, we did not have enough mutations (on average ~1 SNP difference between any pair), and thus do not report these regions' rates. We could also have less power to discover SNPs in annotations with extensive structural variation such as active transposable elements [84]. Transposons, which comprise ~8% of the genome and ~19% of all the SNPs in greenhouse MA lines, had fewer SNPs called than expected in HPG1. This would explain the atypically low transposon substitution rate (Table S3). Therefore, transposon substitution rates in HPG1 cannot be trusted.

7.2.2 Bayesian tip-calibration

For the second approach to estimate a substitution rate, the Bayesian phylogenetics tip-calibration approach, we performed systematic runs and chain convergence assessments of different demographic and molecular clock models. We found the Skygrid demographic model [85] and the lognormal relaxed molecular clock [86] the most appropriate models. Under a relaxed molecular clock, the substitution rate is allowed to vary across branches with a lognormal distribution. The prior used for molecular clock was a Continuous-Time Markov Chain (CTMC) [85,87]. The analysis was carried out remotely at CIPRES PORTAL (v3.1 www.phylo.org) using uninformative priors. The run took about 1,344 CPU hours and performed 1,000 million steps in a Monte Carlo Markov Chain (MCMC), sampling every 100,000 steps. Burn-in was adjusted to 10% of the steps. To visualize the tree output we produced a Maximum Clade Credibility (MCC) tree with a minimum posterior probability threshold of 0.8 and a 10% burn-in using TreeAnnotator (part of BEAST package), and visualized the MCC tree using FigTree (tree.bio.ed.ac.uk/software/figtree/) (Fig. 3B). Additionally, we used DensiTree [88] to simultaneously draw the 10,000 BEAST trees with the highest posterior probability (Fig. 3A). Since all trees were drawn transparently, agreements in both topology and branch lengths appear as densely colored regions, while areas with little agreement appear lighter.

7.2.3 Methylation status of mutated sites

As in many other species, the spectrum of *de novo* mutations in the greenhouse-grown *A. thaliana* MA lines is biased towards G:C→A:T transitions [79], leading to an inflated transition-to-transversion ratio (Ts/Tv). This bias is less pronounced in recent mutations in a Eurasian collection of natural accessions (Fig. 5A of [39] and in HPG1 accessions (Fig. 3D). A recent multigenerational salt stress experiment in the greenhouse also showed a more balanced Ts/Tv [89]. These findings indicate that less benign conditions might promote a lower Ts/Tv, and one possible cause are methylation patterns, known to change under different environments [90].

We interrogated the potential evolutionary role of cytosine methylation in the mutability of cytosine bases in the HPG1 accessions. For reference DNA methylation data, we used previously generated bisulfite-sequencing data of HPG1 strains [78] and of Col-0 MA lines [76], respectively. For both datasets, methylation status was calculated as the fraction of reads with methylated cytosines by the total number of reads at a certain cytosine position in the genome. Our rationale was that if methylation affected mutability, the degree of methylation at positions were we find a new mutation should be higher. To be sure that a given site in HPG1 was a new mutation, we only considered positions for which we could determine that state by alignment to the *A. lyrata* genome [91]. The "tested sites" were positions in HPG1 that had a mutation both from *A. lyrata* and *A. thaliana* Col-0. These positions can be of two kinds, "fixed" if all HPG1 individuals carry the alternative, or "segregating" if both reference and alternative alleles exist in HPG1. As control, "control set", we used cytosine positions that did not vary across HPG1, *A. lyrata* and *A. thaliana*. To produce the methylation distribution of the control set we randomly chose 1,000 invariant cytosine positions. For the test sets, we averaged the methylation degree and compared it with the control distribution.

Ancestral cytosines with higher methylation in both *A. thaliana* Col-0 reference and HPG1 pseudo-reference methylome datasets were more likely to mutate to thymines in HPG1 (Fig. S2 A-D). Additionally, the methylation degree at substitutions inside genes was higher in the HPG1 methylome (Fig. S2 B,D). While some C→T changes could be explained by higher spontaneous deaminations known to happen more often at methylated cytosines, also C→A/G substitutions were more likely to have been methylated. If this process is common enough, the Ts/Tv ratio should decrease. We are far from understanding differences in Ts/Tv in natural and controlled conditions, but definitely methylation status seems to have a strong statistical connection with mutability.

8. Phenotypic association analyses and dating of newly arisen mutations

8.1. Phenotyping

8.1.1 Root

Fifteen root phenotypes were scored for ≥ 10 replicates per genotype over a time-series experiment at the Gregor Mendel Institute in Vienna, using image analysis as described in detail elsewhere [92]. We used the means per genotypes and per time series for association analyses.

8.1.2 Seed size

We spread the seeds of given genotypes on separate plastic square 12 x 12 cm Petri dishes. For faster image acquisition we used a cluster of eight Epson V600 scanners. The scanner cluster was operated by the BRAT Multiscan image acquisition tool (www.gmi.oeaw.ac.at/research-groups/wolfgang-busch/resources/brat/). The resulting 1600 dpi images were analyzed in Fiji software. Scans were converted to 8-bit binary images, thresholded (parameters: setAutoThreshold("Default dark"); setThreshold(20, 255)) and particles analyzed (inclusion parameters: size=0.04-0.25 circularity=0.70-1.00). The 2D seed size was measured in square millimeters (parameters: distance=1600 known=25.4 pixel=1 unit=mm) for 2 plants per genotype, > 500 seeds per plant.

8.1.3 Flowering in the growth chamber

We estimated the flowering time in growth chambers under four vernalization treatments (0, 14, 28 and 63 days of vernalization). We grew 6 replicates per accession divided between two complete randomized blocks for each treatment. Seeds were sown on a 1:1 mixture of Premier Pro-Mix and MetroMix and cold stratified for 6 days (6°C, no light). We then let plants germinate and grow at 18°C, 14 hours of light, 65% humidity. After 3 weeks, we transferred the plants to vernalization conditions (6°C, 8 hours of light, 65% humidity). After vernalization, plants were transferred back to long day conditions. Trays were rotated around the growth chambers every other day throughout the experiment, under both vernalization and ambient conditions. Germination, bolting and flowering dates were recorded every other day until all plants had flowered. Days till flowering or bolting times were calculated from the germination date until the first flower opened and until the first flower bud was developed, respectively. The average flowering time and bolting time per genotype were used for association analyses.

8.1.4 Fecundity in the field

To investigate variation in fecundity in natural conditions, we grew three replicates of each accession in a field experiment following a completely randomized block design. Seeds were sown from 09/20/2012 to 09/22/2012 in 66-well trays (well diameter = 4 cm) on soil from the field site where plants were to be transplanted. The trays were cold stratified for seven days before being placed in a cold frame at the University of Chicago (outdoors, no additional light or heat, but watered as needed and protected from precipitation). Seedlings were transplanted directly into tilled ground at the Warren Wood field station (41.84° N., 86.63° W.), Michigan, USA on 10/13/2012 and 10/14/2012. Seedlings were watered-in and left to overwinter without further intervention. Upon maturation of all fruits, stems were harvested and stored between sheets of newsprint paper. To estimate the fecundity, stems were photographed on a black background and the size of each plant was estimated as the number of pixels occupied by the plant on the image. This measure correlates well with the total length of siliques produced, a classical estimator of fecundity in *A. thaliana* (Spearman's rho=0.84, *p*-value<0.001, data not shown).

8.2 Quantitative genetic analyses

For 63 modern accessions, we measured time to bolting and flowering, seeds per plant, seed size, and 15 root phenotypes in common chamber or common garden settings. For all 100 accessions, climatic information from the bioclim database (www.worldclim.org/bioclim) was extracted using their geographic coordinates. For historic samples, some locations were only known by county name. In this case we assigned the geographic coordinate location of the centroid of the county.

8.2.1 Heritability

We performed association analyses using the R package GenABEL [93], with measured phenotypes (p = 25) and climatic variables (c = 18) as response variables and SNPs as explanatory variables. A Minimum Allele Frequency (MAF) cutoff of 5% was used. The number of assessed SNPs was 391 in a dataset of only modern samples but with imputed genotypes for missing data using Beagle v4.0 [94], and 456 SNPs with a dataset of modern and historic samples, without imputation. For all associations, at least 63 individuals were genotyped for a specific SNP. We first investigated broad sense heritability (H^2) of each trait using ANOVA partition of variance between and within lines using replicates (Table S4). Significance was obtained by common F test in ANOVA. Secondly we used the *polygenic_hglm* function to fit a genome wide kinship matrix to calculate a narrow sense heritability estimate (h^2). This fits a model of the type $y = Zu + \varepsilon$ (see Main text Methods). Significance was calculated employing a likelihood ratio test comparing with a null model. In principle, h^2 is a component of H^2, then its values should theoretically be $h^2 < H^2$. That is not our case. Our

result cannot be interpreted in this framework, since the calculation of both was not done with the same samples: for the h^2 calculation we employed genotype means whereas for the H^2 we used multiple replicated measurements per genotype. The averaging of replicates per genotype in h^2 reduced environmental and developmental noise and thus we would expect $h^2 > H^2$. We did this so the climatic estimates of h^2, for which we only have one value per genotype, would be comparable with the phenotypic h^2 ones (Table S4).

8.2.2 Linear Models

For association analyses we first employed a linear mixed model that fitted the kinship matrix using the *mmscore* function. This model is of the type: $y = Xb + Zu + \varepsilon$ (see Main text Methods) [95]. Only three significant SNP hits were discovered using a 5% significance threshold after False Discovery Rate correction (FDR). This was expected since we have few variants and these would have originated in an approximated phylogeny structure. We concluded that fitting the kinship matrix in our model was not appropriate since there would be no residual variation for association with specific SNPs. With this rationale we employed a fixed effects linear model using the *qtscore* function [96]. This model is of the type: $y = Xb + \varepsilon$; where no random effect of genome background is fit. To reduce the risk of having false-positives, we took a conservative permutation strategy by carrying out association with over 1,000 randomized datasets (permuting phenotypes across individuals) and used the resulting empirical p-value distribution to correct p-values estimated with the original dataset. SNPs with p-values below 5% in the empirical p-value distribution should be considered significant (but see next section). In climatic models, we included longitude and latitude as covariates to correct for any spurious association between SNPs and climate gradients created by the migratory pattern of isolation by distance.

8.2.3 Evaluation of significance

Significant SNPs were interspersed throughout the genome (Fig. 4) and their p-values and phenotypic effects did not correlate with the minimum age of the SNPs nor with their allele frequency, something that could have indicated that the significance was merely driven by the higher statistical power of intermediate frequency variants. Using QQ plots to assess inflation or deflation of p-values, we observed generally that permutation corrected p-values were deflated — another evidence of our conservative strategy. Straight horizontal series of points in QQ plots indicate that multiple SNPs have identical p-values, a pattern that we attributed to long range LD, i.e. lack of independence (see Text S2 for trait distributions and QQ plots from each association analysis).

To further ensure that we avoided false positive results, we also prioritized SNPs whose empirical p-value was not below 5% only but also below 5% / (number of SNPs + number of traits) = 0.01%. This "double" Bonferroni correction was very conservative (Table 1, Table S5).

8.2.4 Context of de novo mutations associated with phenotypes

For each SNP in our dataset, we determined the ancestral and derived states, by identifying which allele was found in the oldest herbarium samples. We compared the time of emergence and the centroid of geographic distribution of the alternative alleles of SNP hits to random draws of SNPs with the same MAF filtering (5%) (Fig. S1).

8.2.5 Functional information

On top of phenotypic and climatic associations of SNP hits, we also provide a likely functional effect employing a commonly used amino acid matrix of biochemical effects [45]. Functional information of gene name and ontology categorization of SNP hits was obtained from www.arabidopsis.org/portals/genAnnotation/gene_structural_annotation/annotation_data.jsp and www.arabidopsis.org/tools/bulk/go/ (Table 1 and Table S5).

8.2.6 Proof of concept examples

We argue that the power of our association approach relies on the fact that HPG1 lines resemble Near Isogenic Lines (NILs) produced by experimental crosses [97] (Fig. S2A). Similar to genome-wide association studies (GWA), power depends on many factors, namely the noise of phenotype under study, architecture of phenotypic trait, quality of genotyping, population structure, sample diversity, sample size, allele frequency, and recombination. On one hand, association analyses in NILs suffer from large linkage blocks, but confident results can be achieved due to accurate measurement of phenotypes, limited genetic differences between any two lines, and high quality genotypes. In common GWA studies such as in humans, there are multiple confounding effects. Among the confounders are (1) that any two samples differ in hundreds of thousands of SNPs, and (2) that historical and geographic stratification produce non-random correlations among those SNP differences. This considerably complicates the identification of phenotypic effects at specific genes, and power relies greatly on large sample sizes to achieve the sufficient number of recombination between markers.

To provide support for the non-synonymous SNP on chromosome 5, at position 6,508,329 in AT5G19330, we looked for pairs of lines that carry the ancestral and the derived allele, but that differ in few (or no other) SNPs in the genome. When considering all genic substitutions with a minimum allele frequency of 5% (Fig. S2A), we identified 20 pairs of lines differing only in the

AT5G19330 SNP and another linked SNP (located on a different chromosome, association p-value > 0.4). The phenotypic differences in mean gravitropic score of these almost-identical pairs were significantly higher than phenotypic differences among all pairs of HPG1 lines, and genetically identical pairs attending to substitutions inside genes (Fig. S2A). Furthermore, this SNP was not in complete linkage with any other SNP hit ($r^2 < 0.5$) (Fig. S2D). The same approach was used to examine the SNPs in AT1G54440 (Fig. S2E) and AT2G16580 (Fig. S2F), which represent an intermediate and a high LD example.

References

74. Weiß CL, Schuenemann VJ, Devos J, Shirsekar G, Reiter E, Gould BA, et al. Temporal patterns of damage and decay kinetics of DNA retrieved from plant herbarium specimens. Royal Society Open Science. The Royal Society; 2016;3: 160239. doi:10.1098/rsos.160239

75. Meyer M, Kircher M. Illumina sequencing library preparation for highly multiplexed target capture and sequencing. Cold Spring Harb Protoc. 2010;2010: db.prot5448. doi:10.1101/pdb.prot5448

76. Becker C, Hagmann J, Müller J, Koenig D, Stegle O, Borgwardt K, et al. Spontaneous epigenetic variation in the Arabidopsis thaliana methylome. Nature. nature.com; 2011;480: 245–249. doi:10.1038/nature10555

77. Shaw RG, Byers DL, Darmo E. Spontaneous mutational effects on reproductive traits of arabidopsis thaliana. Genetics. 2000;155: 369–378. Available: http://www.ncbi.nlm.nih.gov/pubmed/10790410

78. Hagmann J, Becker C, Müller J, Stegle O, Meyer RC, Wang G, et al. Century-scale methylome stability in a recently diverged Arabidopsis thaliana lineage. PLoS Genet. 2015;11: e1004920–e1004920. doi:10.1371/journal.pgen.1004920

79. Ossowski S, Schneeberger K, Lucas-Lledó JI, Warthmann N, Clark RM, Shaw RG, et al. The rate and molecular spectrum of spontaneous mutations in Arabidopsis thaliana. Science. 2010;327: 92–94. doi:10.1126/science.1180677

80. Platt A, Horton M, Huang YS, Li Y, Anastasio AE, Mulyati NW, et al. The scale of population structure in Arabidopsis thaliana. PLoS Genet. 2010;6: e1000843. doi:10.1371/journal.pgen.1000843

81. Wolfe KH, Sharp PM, Li W-H. Rates of synonymous substitution in plant nuclear genes. J Mol Evol. Springer-Verlag; 1989;29: 208–211. doi:10.1007/BF02100204

82. 1001 Genomes Consortium. 1,135 Genomes Reveal the Global Pattern of Polymorphism in Arabidopsis thaliana. Cell. Elsevier; 2016;166: 481–491. doi:10.1016/j.cell.2016.05.063

83. Librado P, Rozas J. DnaSP v5: a software for comprehensive analysis of DNA polymorphism data. Bioinformatics. 2009;25: 1451–1452. doi:10.1093/bioinformatics/btp187

84. Treangen TJ, Salzberg SL. Repetitive DNA and next-generation sequencing: computational challenges and solutions. Nat Rev Genet. 2012;13: 36–46. doi:10.1038/nrg3117

85. Gill MS, Lemey P, Faria NR, Rambaut A, Shapiro B, Suchard M a. Improving Bayesian population dynamics inference: a coalescent-based model for multiple loci. Mol Biol Evol. 2012;30: 713–724. doi:10.1093/molbev/mss265

86. Drummond AJ, Ho SYW, Phillips MJ, Rambaut A. Relaxed phylogenetics and dating with

confidence. PLoS Biol. 2006;4: e88–e88. doi:10.1371/journal.pbio.0040088

87. Ferreira M a. R, Suchard M a. Bayesian analysis of elapsed times in continuous-time Markov chains. Can J Stat. 2008;36: 355–368. doi:10.1002/cjs.5550360302

88. Bouckaert RR. DensiTree: making sense of sets of phylogenetic trees. Bioinformatics. 2010;26: 1372–1373. doi:10.1093/bioinformatics/btq110

89. Jiang C, Mithani A, Belfield EJ, Mott R, Hurst LD, Harberd NP. Environmentally responsive genome-wide accumulation of de novo Arabidopsis thaliana mutations and epimutations. Genome Res. 2014;24: 1821–1829. doi:10.1101/gr.177659.114

90. Wibowo A, Becker C, Marconi G, Durr J, Price J, Hagmann J, et al. Hyperosmotic stress memory in Arabidopsis is mediated by distinct epigenetically labile sites in the genome and is restricted in the male germline by DNA glycosylase activity. eLife. eLife Sciences Publications Limited; 2016;5: e13546. doi:10.7554/eLife.13546

91. Hu TT, Pattyn P, Bakker EG, Cao J, Cheng J-F, Clark RM, et al. The Arabidopsis lyrata genome sequence and the basis of rapid genome size change. Nat Genet. Nature Publishing Group; 2011;43: 476–481. doi:10.1038/ng.807

92. Slovak R, Göschl C, Su X, Shimotani K, Shiina T, Busch W. A scalable open-source pipeline for large-scale root phenotyping of Arabidopsis. Plant Cell. 2014;26: 2390–2403. doi:10.1105/tpc.114.124032

93. Aulchenko YS, Ripke S, Isaacs A, van Duijn CM. GenABEL: an R library for genome-wide association analysis. Bioinformatics. 2007;23: 1294–1296. doi:10.1093/bioinformatics/btm108

94. Browning BL, Browning SR. Genotype Imputation with Millions of Reference Samples. Am J Hum Genet. 2016;98: 116–126. doi:10.1016/j.ajhg.2015.11.020

95. Wilson AJ, Réale D, Clements MN, Morrissey MM, Postma E, Walling CA, et al. An ecologist's guide to the animal model. J Anim Ecol. 2010;79: 13–26. doi:10.1111/j.1365-2656.2009.01639.x

96. Aulchenko YS, de Koning D-J, Haley C. Genomewide rapid association using mixed model and regression: a fast and simple method for genomewide pedigree-based quantitative trait loci association analysis. Genetics. 2007;177: 577–585. doi:10.1534/genetics.107.075614

97. Weigel D. Natural variation in Arabidopsis: from molecular genetics to ecological genomics. Plant Physiol. 2012;158: 2–22. doi:10.1104/pp.111.189845
