# Supplementary material for: The rate and potential relevance of new mutations in a colonizing plant lineage
Source: PLoS Genet. 2018 Feb 12;14(2):e1007155. doi: 10.1371/journal.pgen.1007155 (PMC5825158; doi:10.1371/journal.pgen.1007155)
Supplement: S2 Text — (PDF) [file pgen.1007155.s002.pdf]

Text S2

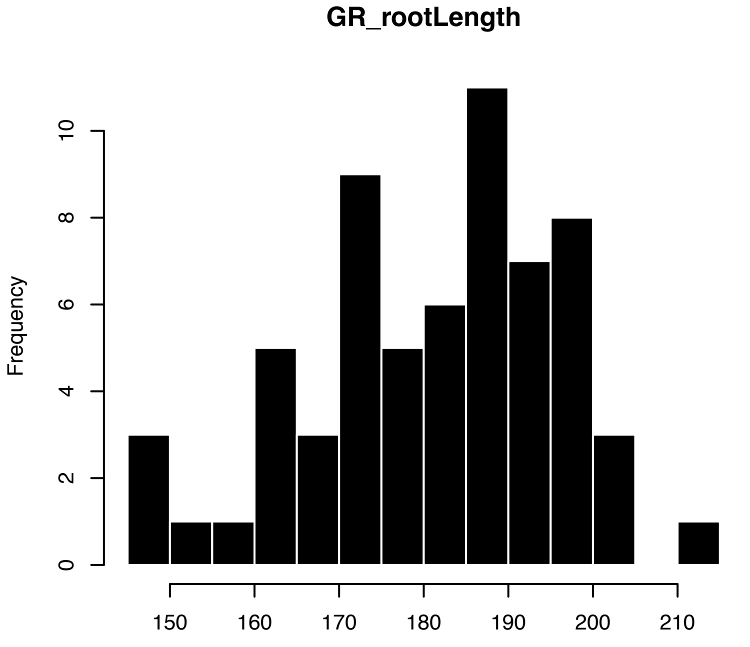
GR_rootLength
Frequency
0 2 4 6 8 10
150 160 170 180 190 200 210

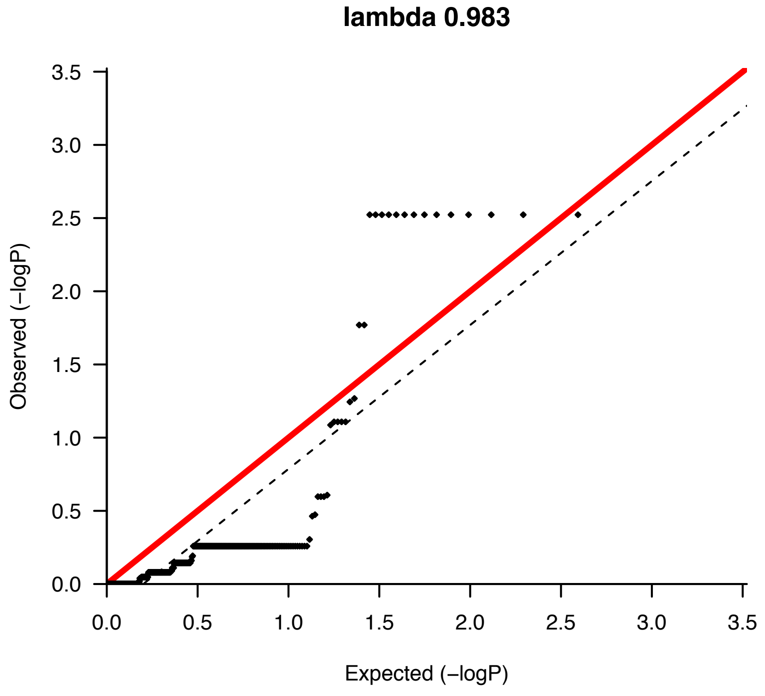
lambda 0.983
Observed (−logP)
Expected (−logP)
0.0 0.5 1.0 1.5 2.0 2.5 3.0 3.5

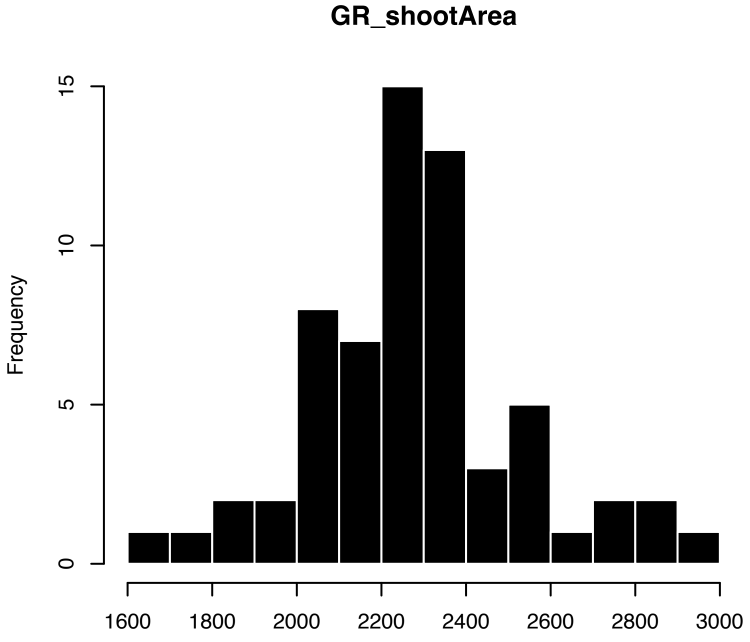
GR_shootArea
Frequency
0 5 10 15
1600 1800 2000 2200 2400 2600 2800 3000

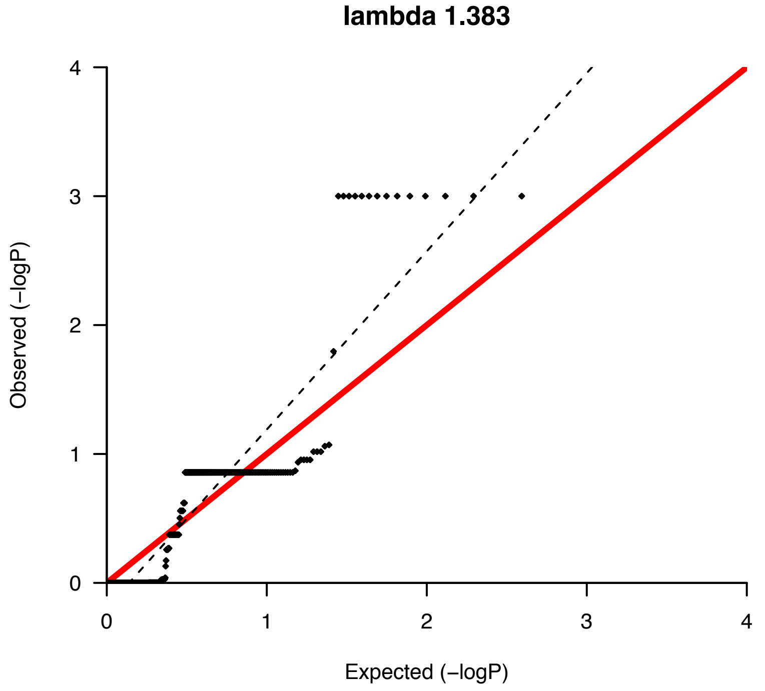
lambda 1.383
Observed (−logP)
Expected (−logP)
0 1 2 3 4

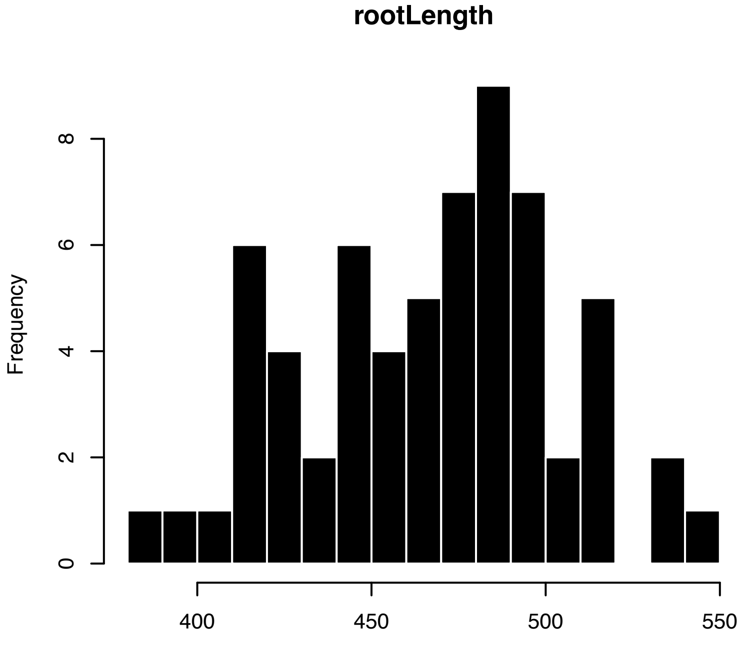
rootLength
Frequency
0 2 4 6 8
400 450 500 550

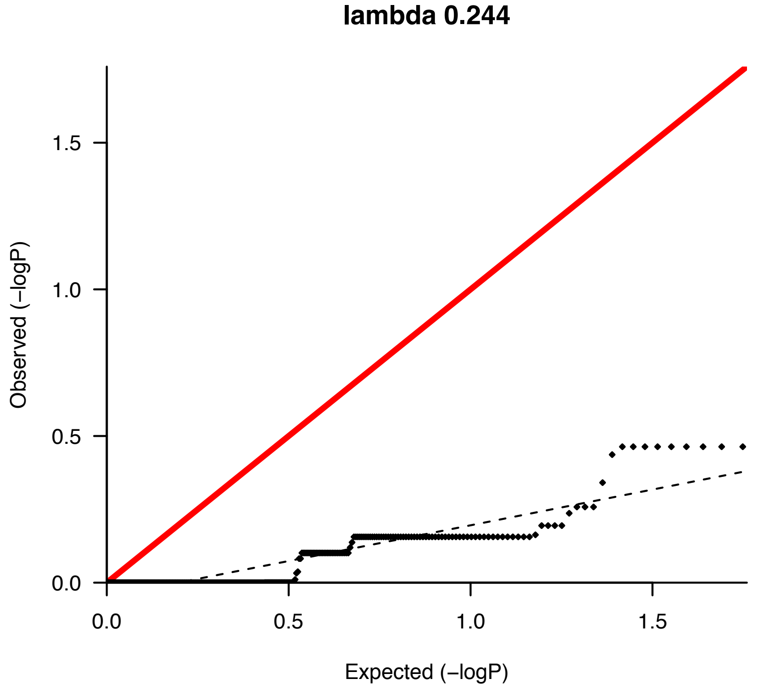
lambda 0.244
Observed (−logP)
Expected (−logP)
0.0 0.5 1.0 1.5

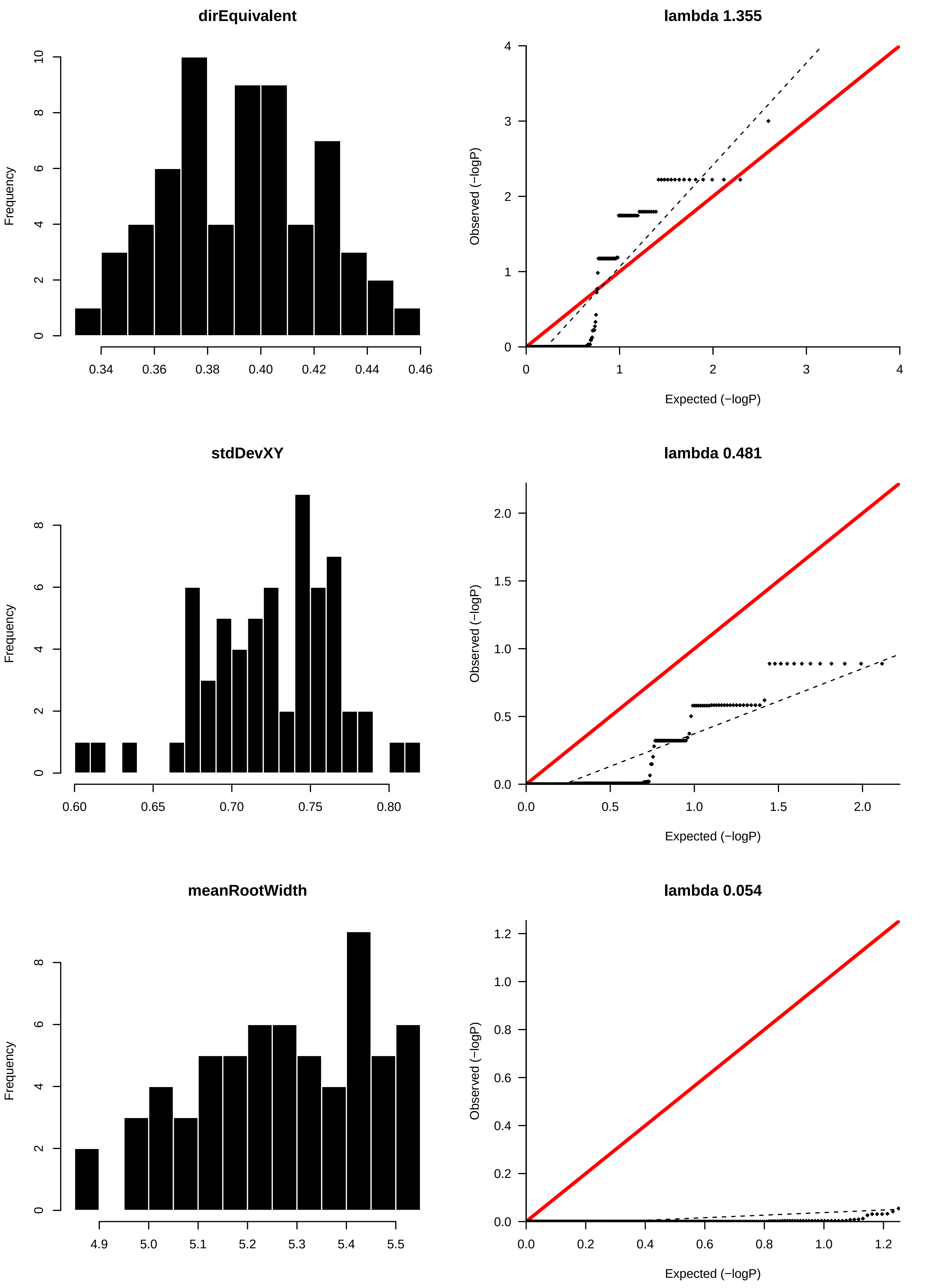
dirEquivalent
Frequency
0 2 4 6 8 10
0.34 0.36 0.38 0.40 0.42 0.44 0.46
lambda 1.355
Observed (−logP)
Expected (−logP)
0 1 2 3 4
stdDevXY
Frequency
0 2 4 6 8
0.60 0.65 0.70 0.75 0.80
lambda 0.481
Observed (−logP)
Expected (−logP)
0.0 0.5 1.0 1.5 2.0
meanRootWidth
Frequency
0 2 4 6 8
4.9 5.0 5.1 5.2 5.3 5.4 5.5
lambda 0.054
Observed (−logP)
Expected (−logP)
0.0 0.2 0.4 0.6 0.8 1.0 1.2

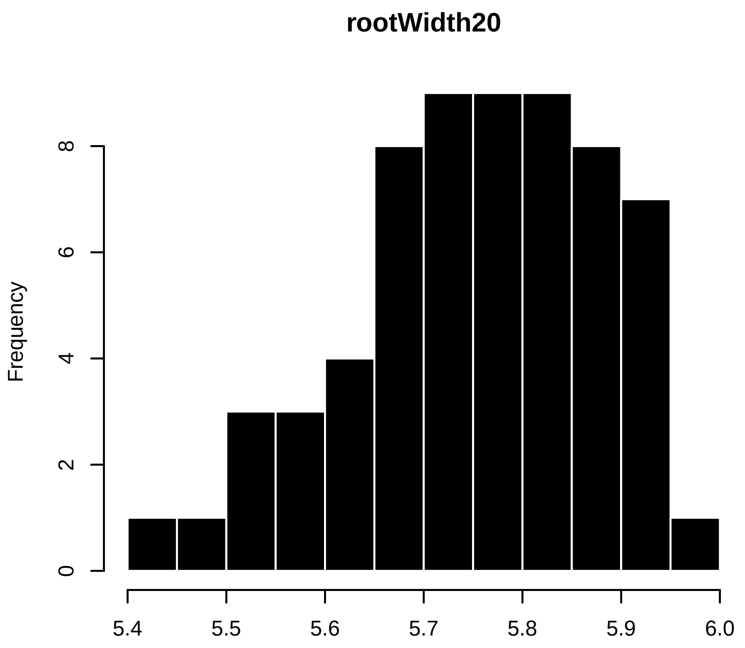
rootWidth20
Frequency
0
2
4
6
8
5.4
5.5
5.6
5.7
5.8
5.9
6.0

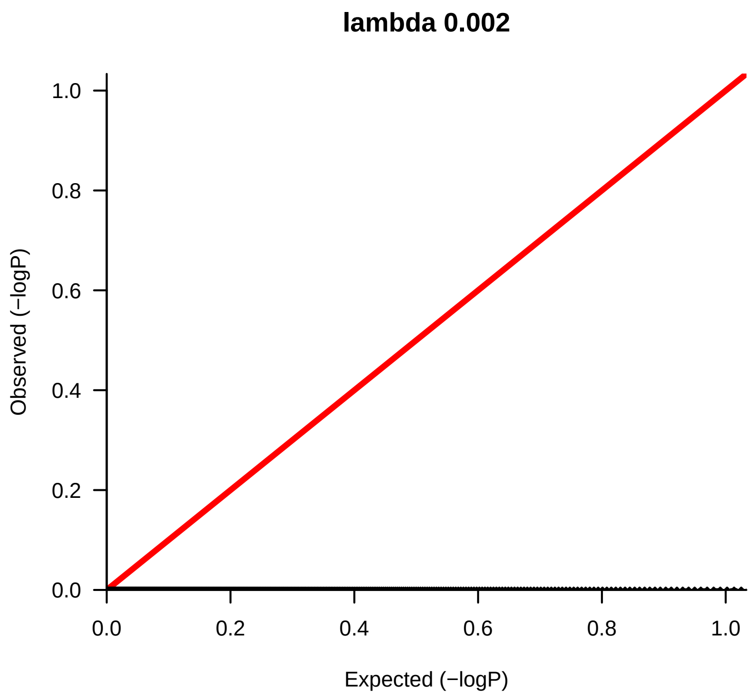
lambda 0.002
Observed (−logP)
Expected (−logP)
0.0
0.2
0.4
0.6
0.8
1.0

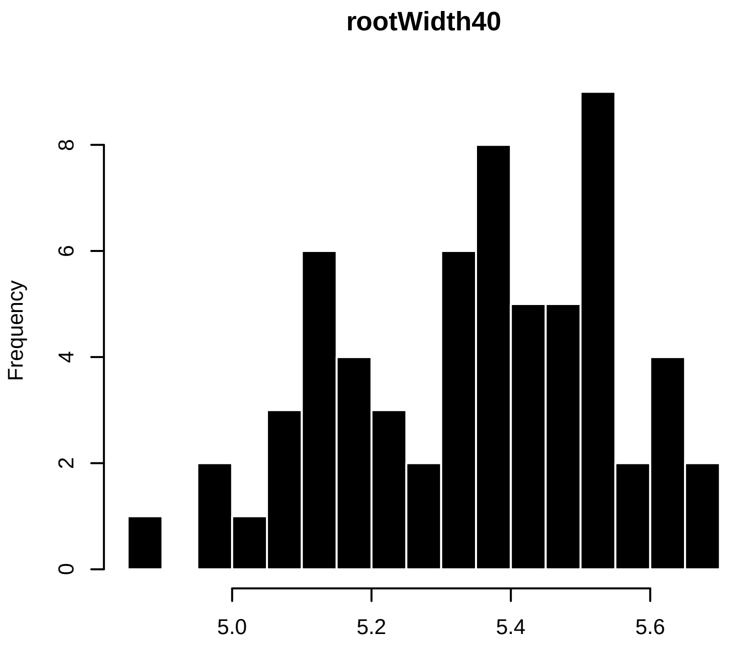
rootWidth40
Frequency
0
2
4
6
8
5.0
5.2
5.4
5.6

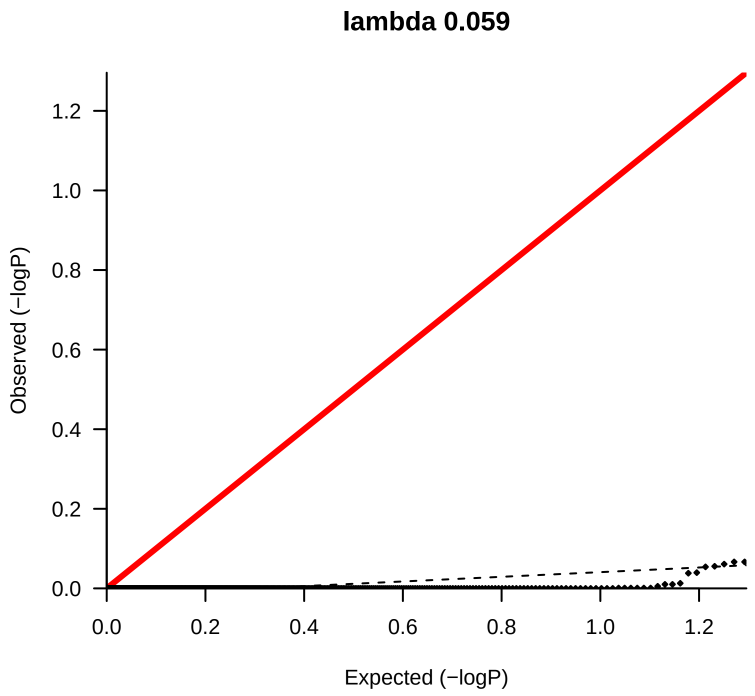
lambda 0.059
Observed (−logP)
Expected (−logP)
0.0
0.2
0.4
0.6
0.8
1.0
1.2

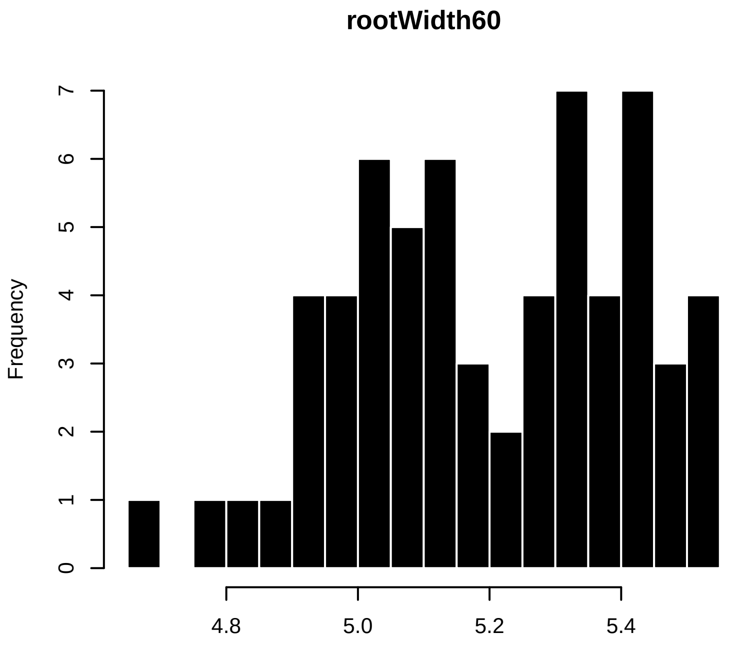
rootWidth60
Frequency
0
1
2
3
4
5
6
7
4.8
5.0
5.2
5.4

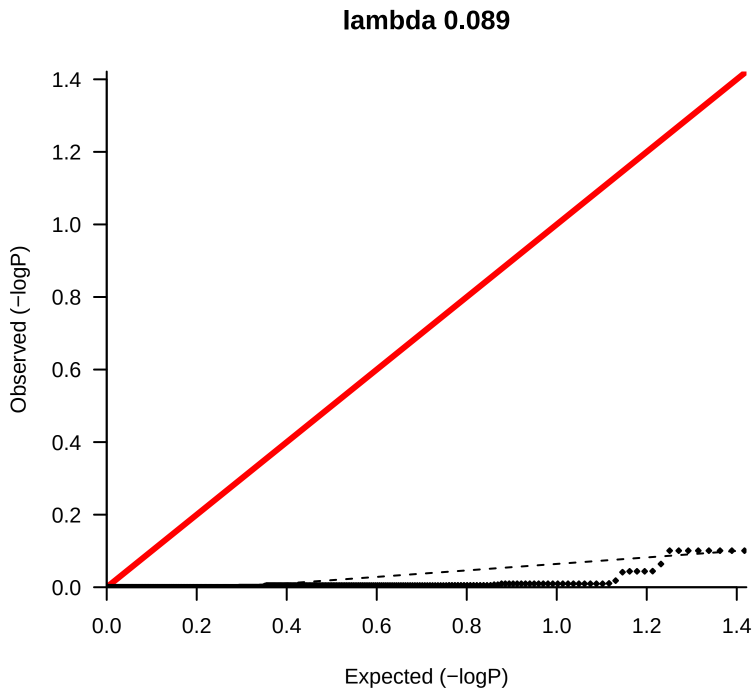
lambda 0.089
Observed (−logP)
Expected (−logP)
0.0
0.2
0.4
0.6
0.8
1.0
1.2
1.4

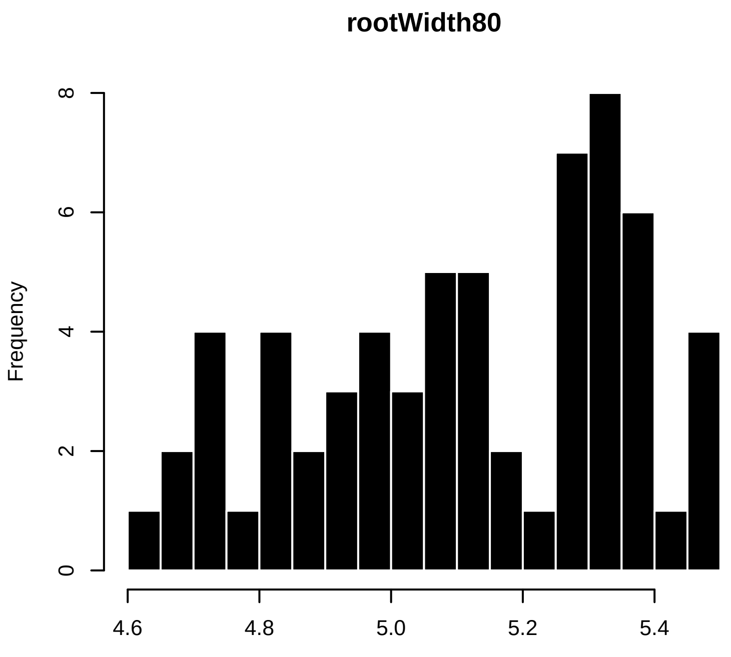
rootWidth80
Frequency
0
2
4
6
8
4.6
4.8
5.0
5.2
5.4

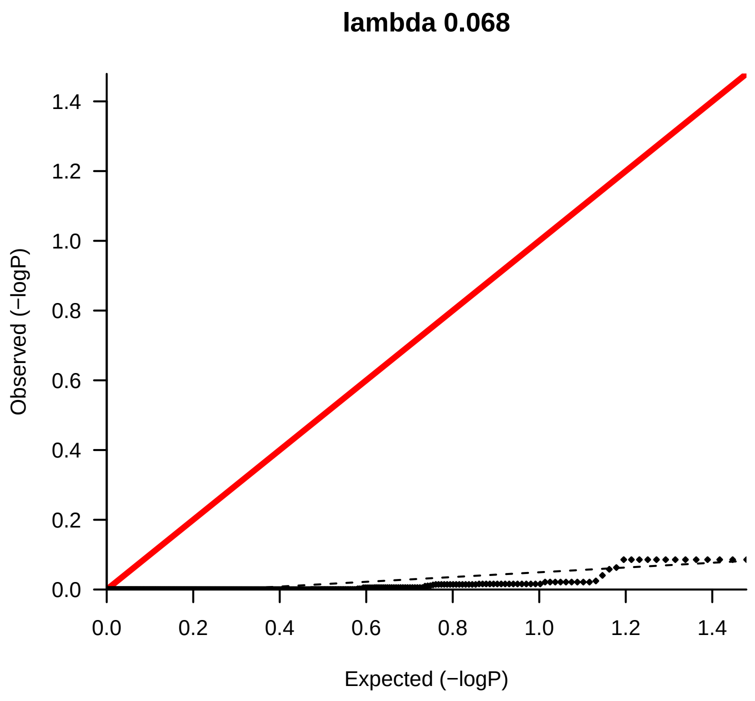
lambda 0.068
Observed (−logP)
Expected (−logP)
0.0
0.2
0.4
0.6
0.8
1.0
1.2
1.4

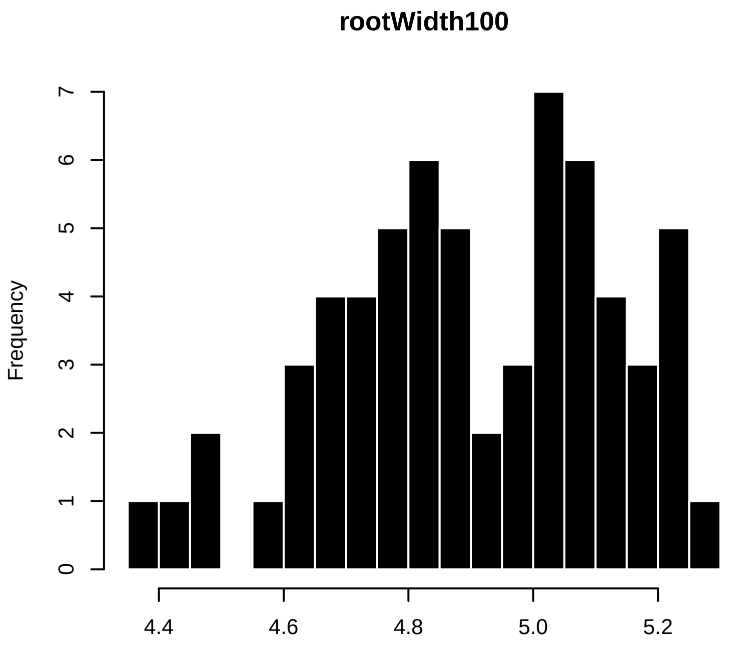
rootWidth100
Frequency
0
1
2
3
4
5
6
7
4.4
4.6
4.8
5.0
5.2

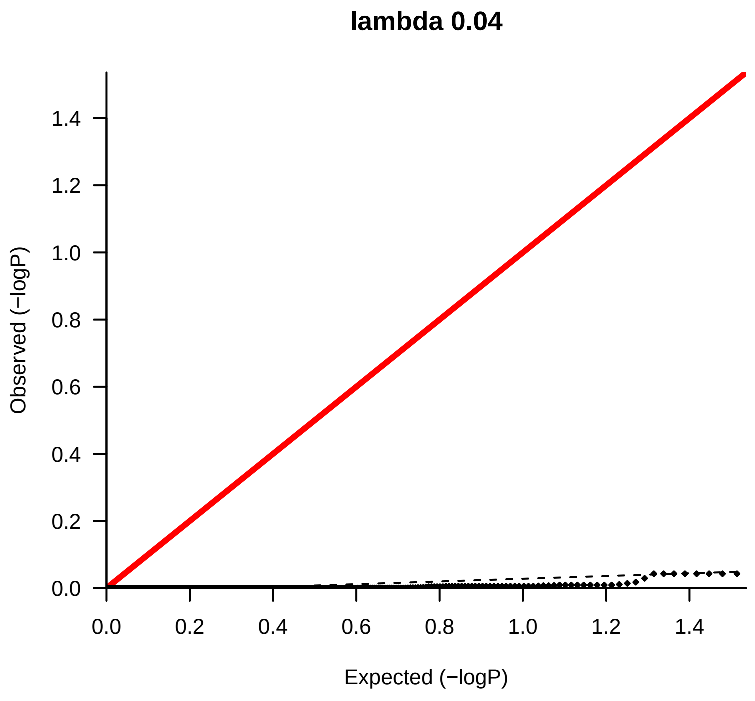
lambda 0.04
Observed (−logP)
Expected (−logP)
0.0
0.2
0.4
0.6
0.8
1.0
1.2
1.4

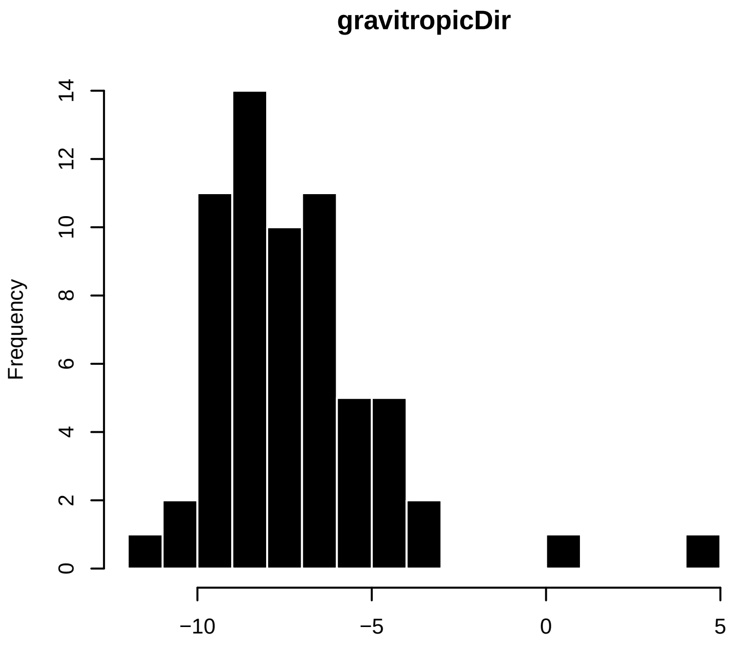
gravitropicDir
Frequency
0
2
4
6
8
10
12
14
−10
−5
0
5

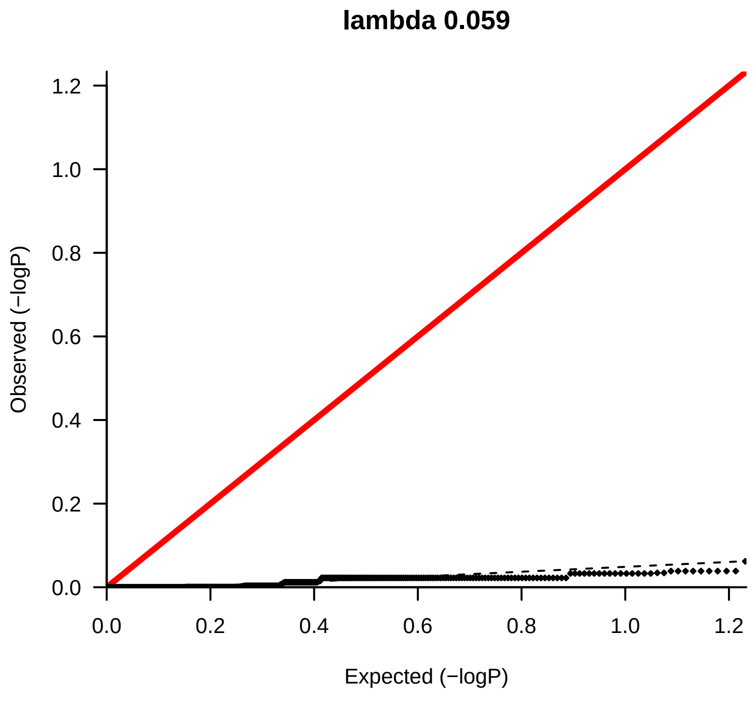
lambda 0.059
Observed (−logP)
Expected (−logP)
0.0
0.2
0.4
0.6
0.8
1.0
1.2

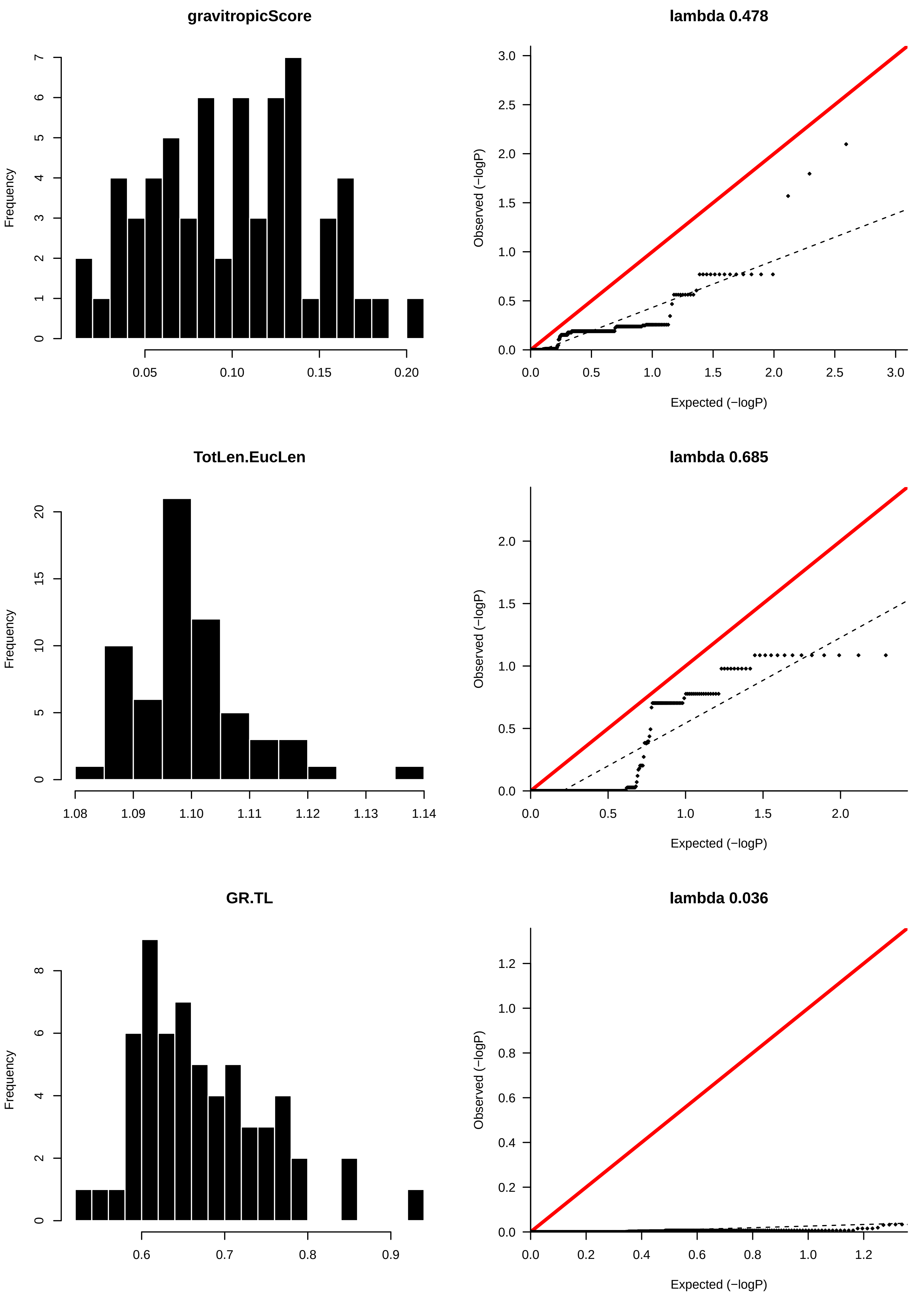
gravitropicScore
Frequency
lambda 0.478
Observed (−logP)
Expected (−logP)
TotLen.EucLen
Frequency
lambda 0.685
Observed (−logP)
Expected (−logP)
GR.TL
Frequency
lambda 0.036
Observed (−logP)
Expected (−logP)

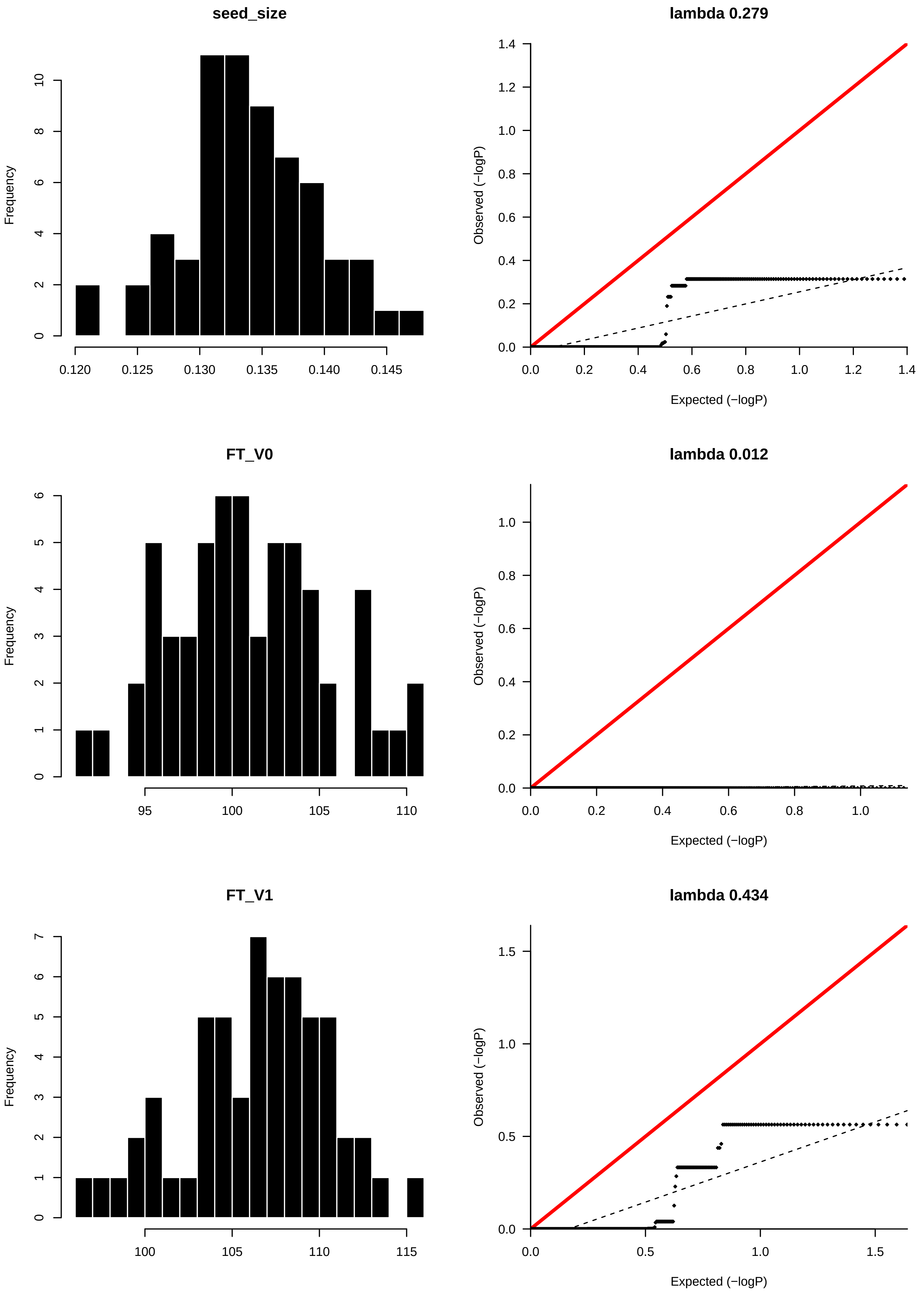
seed_size
Frequency
0.120
0.125
0.130
0.135
0.140
0.145
lambda 0.279
Observed (−logP)
Expected (−logP)
FT_V0
Frequency
95
100
105
110
lambda 0.012
Observed (−logP)
Expected (−logP)
FT_V1
Frequency
100
105
110
115
lambda 0.434
Observed (−logP)
Expected (−logP)

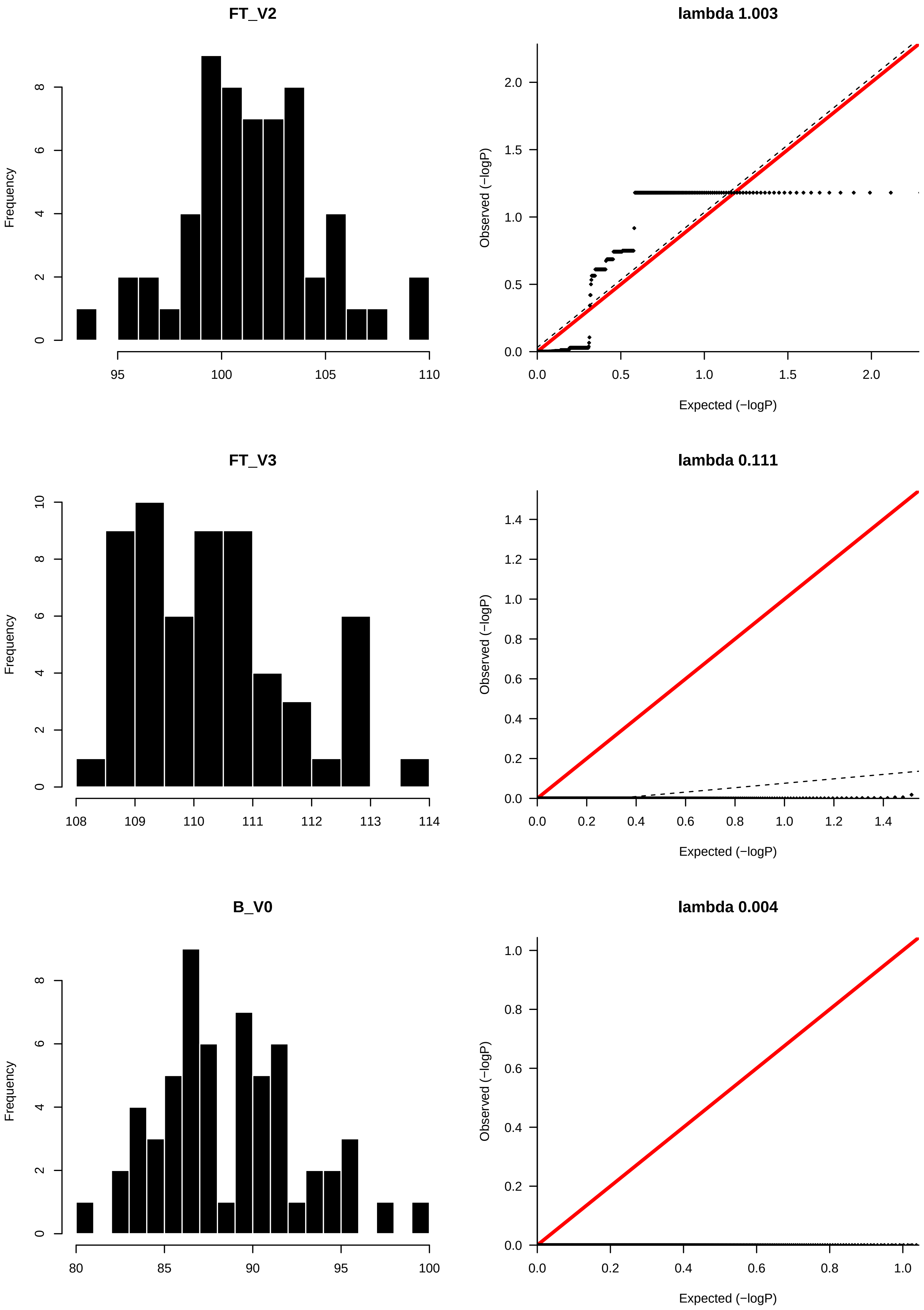

FT_V2
Frequency
0 2 4 6 8
95 100 105 110
lambda 1.003
Observed (−logP)
0.0 0.5 1.0 1.5 2.0
Expected (−logP)
0.0 0.5 1.0 1.5 2.0
FT_V3
Frequency
0 2 4 6 8 10
108 109 110 111 112 113 114
lambda 0.111
Observed (−logP)
0.0 0.2 0.4 0.6 0.8 1.0 1.2 1.4
Expected (−logP)
0.0 0.2 0.4 0.6 0.8 1.0 1.2 1.4
B_V0
Frequency
0 2 4 6 8
80 85 90 95 100
lambda 0.004
Observed (−logP)
0.0 0.2 0.4 0.6 0.8 1.0
Expected (−logP)
0.0 0.2 0.4 0.6 0.8 1.0

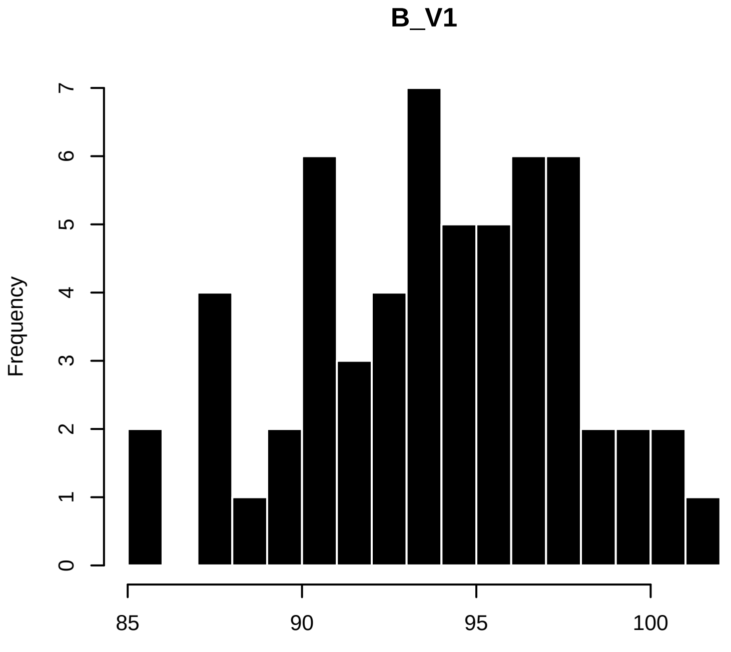
B_V1
Frequency
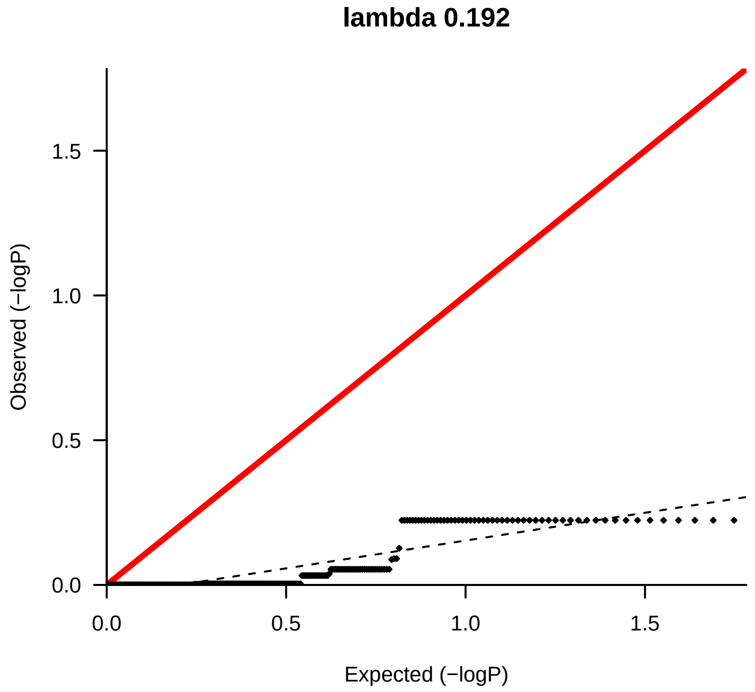
lambda 0.192
Observed (−logP)
Expected (−logP)
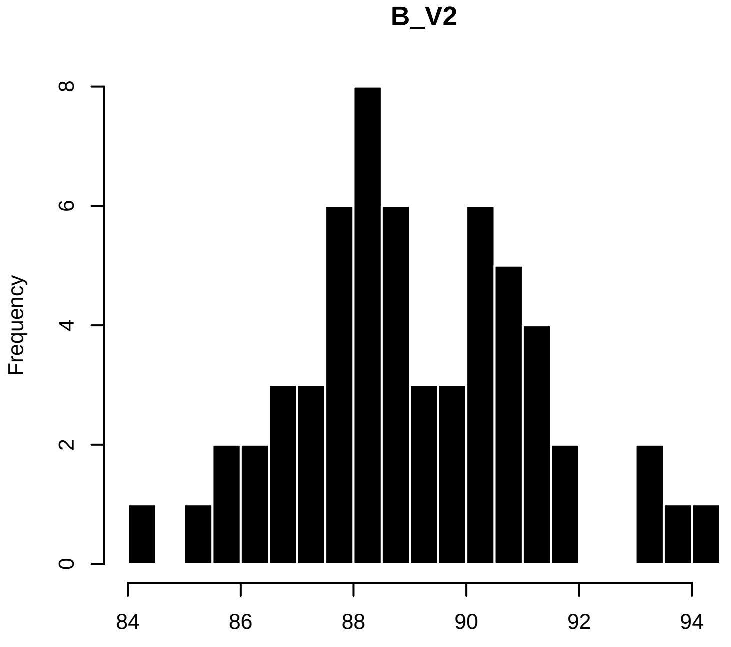
B_V2
Frequency
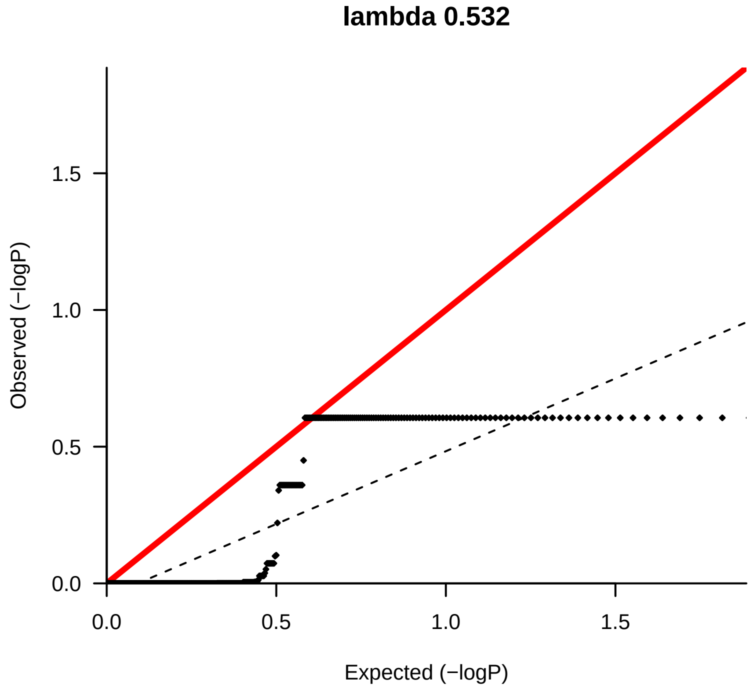
lambda 0.532
Observed (−logP)
Expected (−logP)
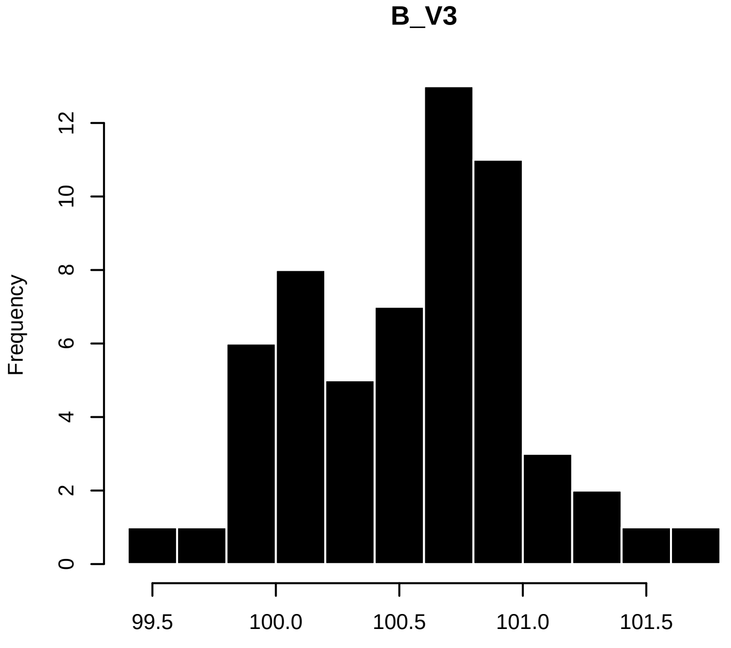
B_V3
Frequency
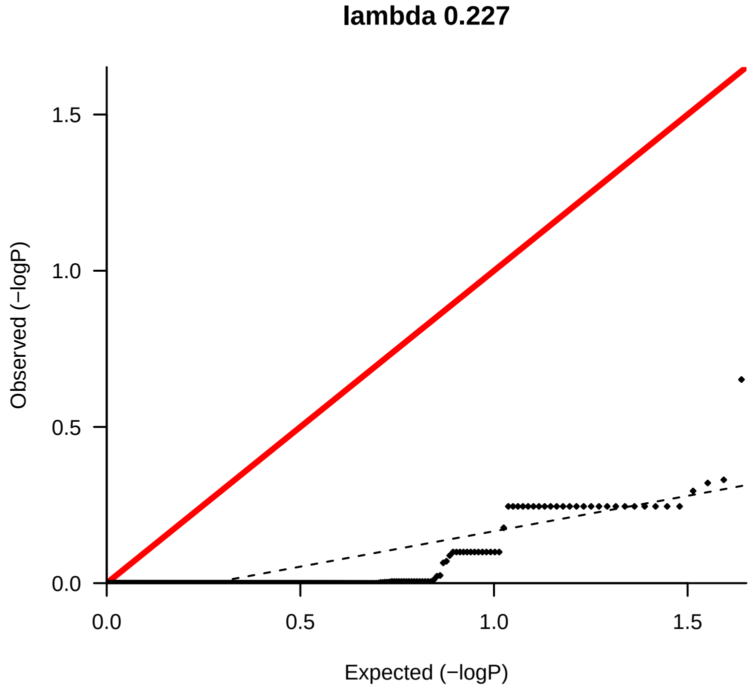
lambda 0.227
Observed (−logP)
Expected (−logP)

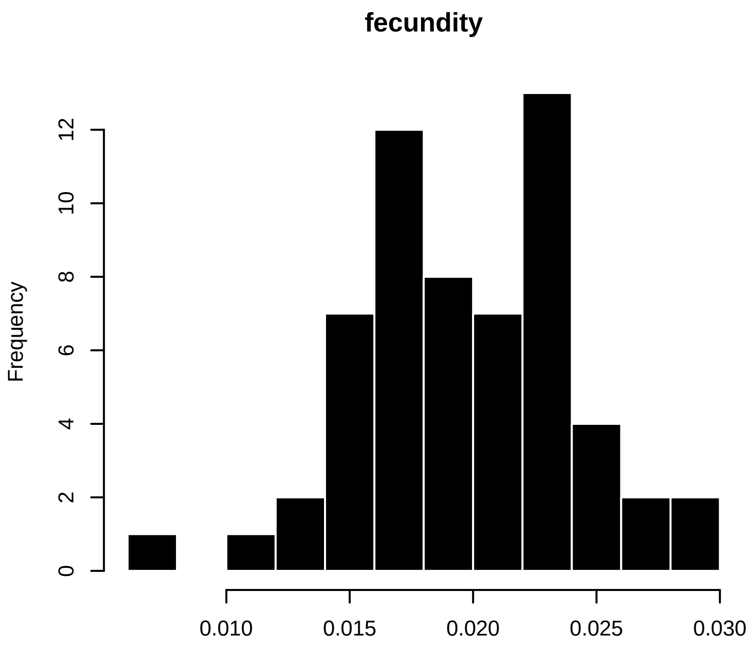
fecundity
Frequency
0
2
4
6
8
10
12
0.010
0.015
0.020
0.025
0.030

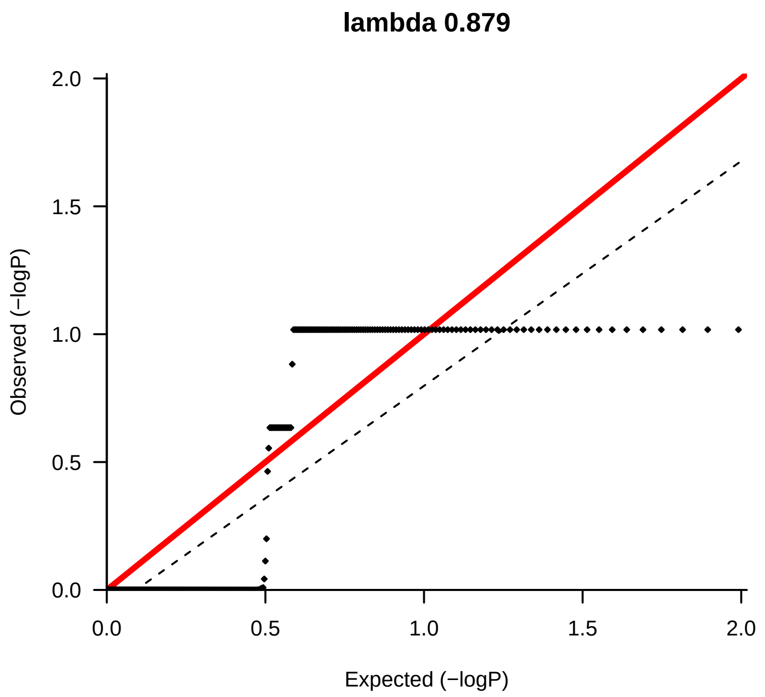
lambda 0.879
Observed (−logP)
Expected (−logP)
0.0
0.5
1.0
1.5
2.0

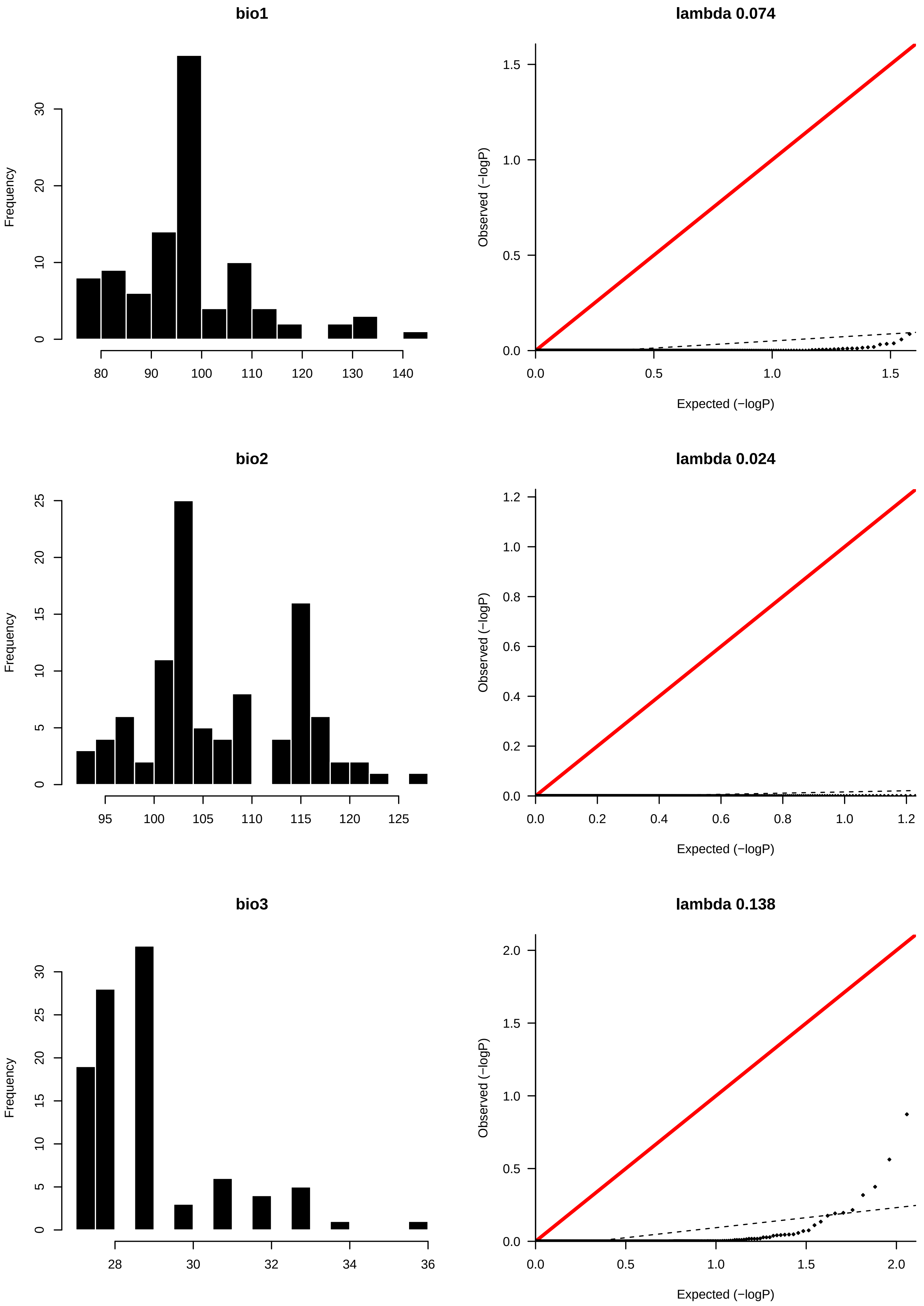
bio1
Frequency
0
10
20
30
80
90
100
110
120
130
140
lambda 0.074
Observed (−logP)
Expected (−logP)
0.0
0.5
1.0
1.5
bio2
Frequency
0
5
10
15
20
25
95
100
105
110
115
120
125
lambda 0.024
Observed (−logP)
Expected (−logP)
0.0
0.2
0.4
0.6
0.8
1.0
1.2
bio3
Frequency
0
5
10
15
20
25
30
28
30
32
34
36
lambda 0.138
Observed (−logP)
Expected (−logP)
0.0
0.5
1.0
1.5
2.0

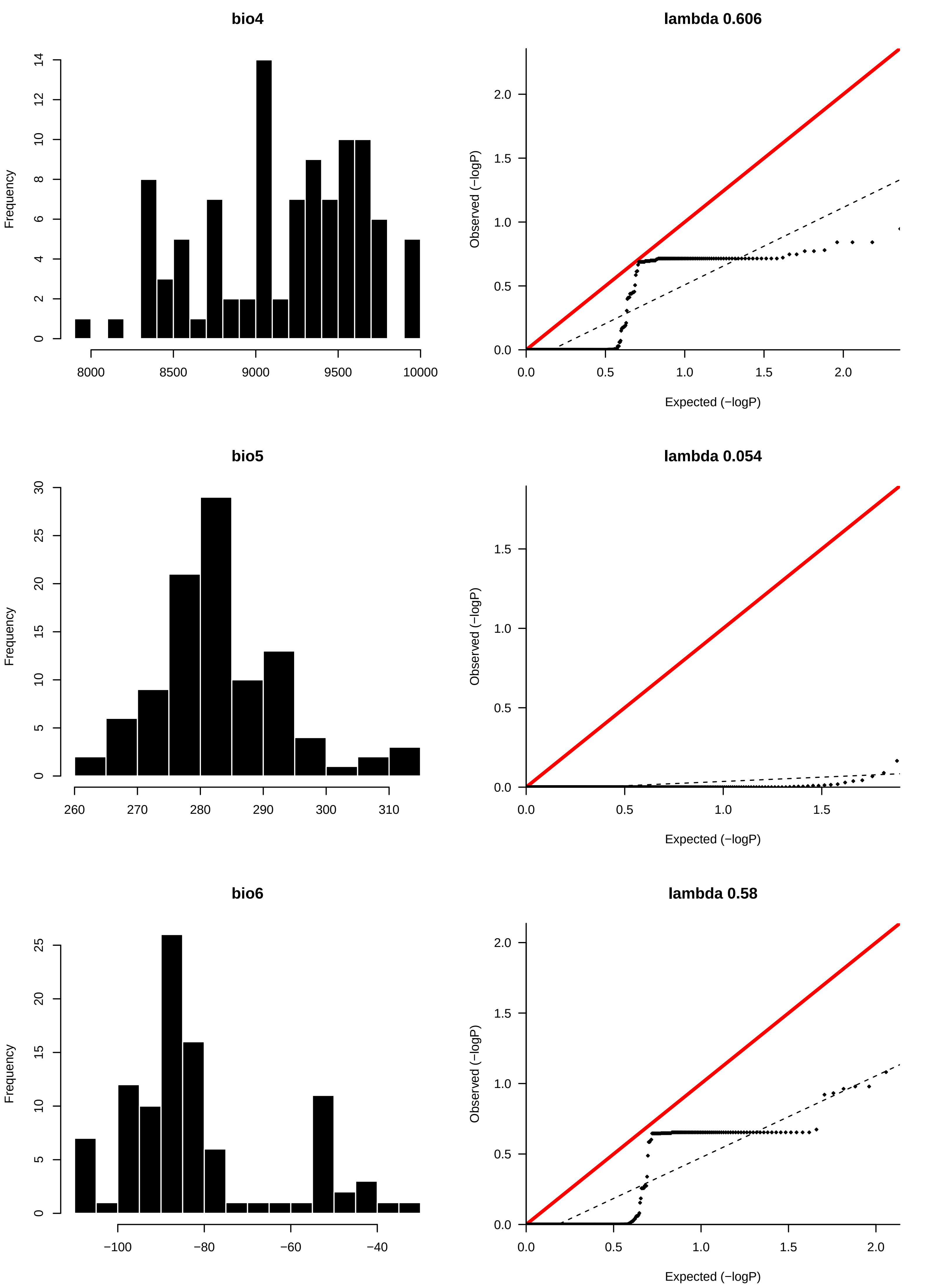

bio4
Frequency
0
2
4
6
8
10
12
14
8000
8500
9000
9500
10000
lambda 0.606
Observed (−logP)
Expected (−logP)
0.0
0.5
1.0
1.5
2.0
bio5
Frequency
0
5
10
15
20
25
30
260
270
280
290
300
310
lambda 0.054
Observed (−logP)
Expected (−logP)
0.0
0.5
1.0
1.5
bio6
Frequency
0
5
10
15
20
25
−100
−80
−60
−40
lambda 0.58
Observed (−logP)
Expected (−logP)
0.0
0.5
1.0
1.5
2.0

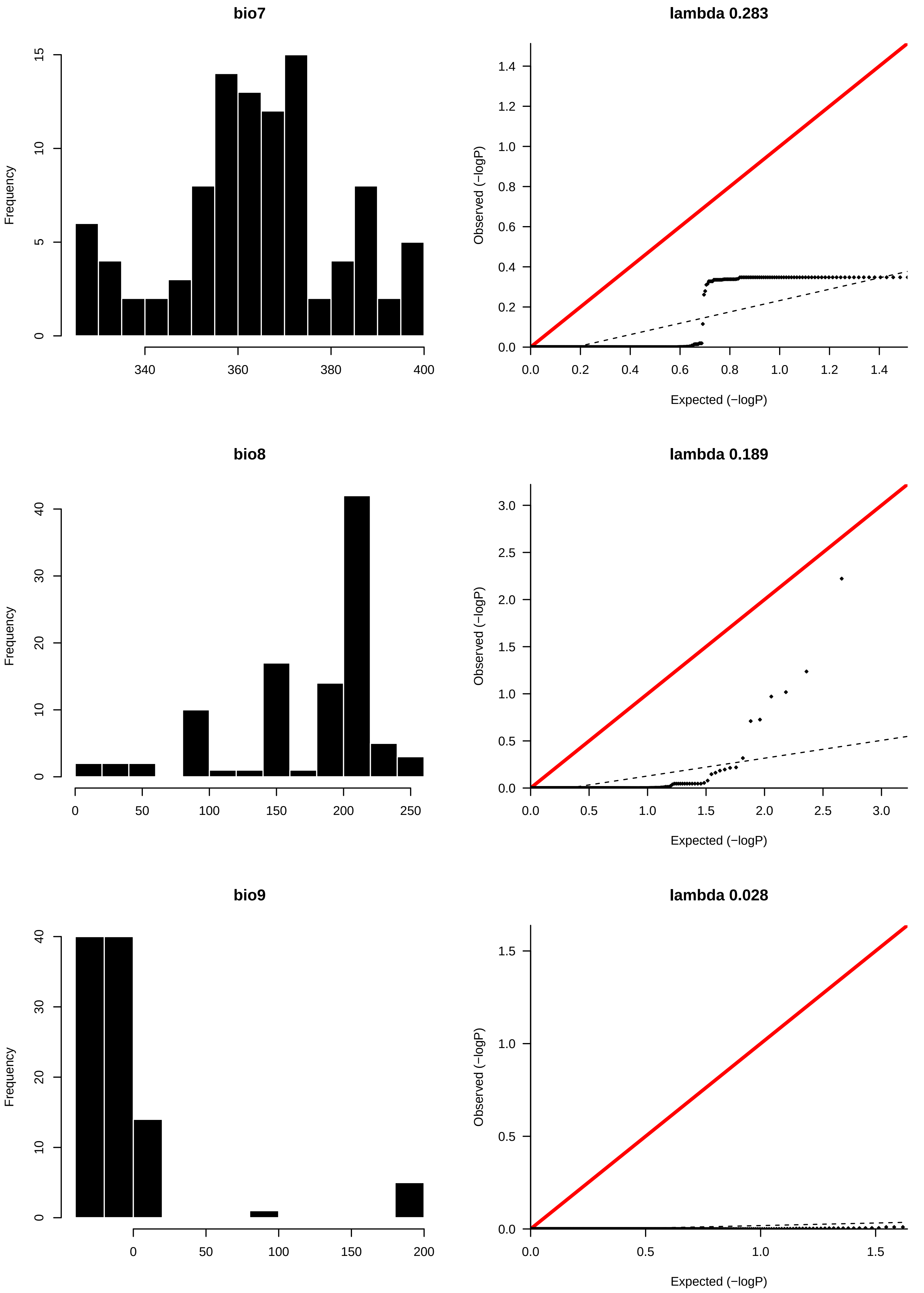
bio7
Frequency
0
5
10
15
340
360
380
400
lambda 0.283
Observed (−logP)
Expected (−logP)
0.0
0.2
0.4
0.6
0.8
1.0
1.2
1.4
bio8
Frequency
0
10
20
30
40
0
50
100
150
200
250
lambda 0.189
Observed (−logP)
Expected (−logP)
0.0
0.5
1.0
1.5
2.0
2.5
3.0
bio9
Frequency
0
10
20
30
40
0
50
100
150
200
lambda 0.028
Observed (−logP)
Expected (−logP)
0.0
0.5
1.0
1.5

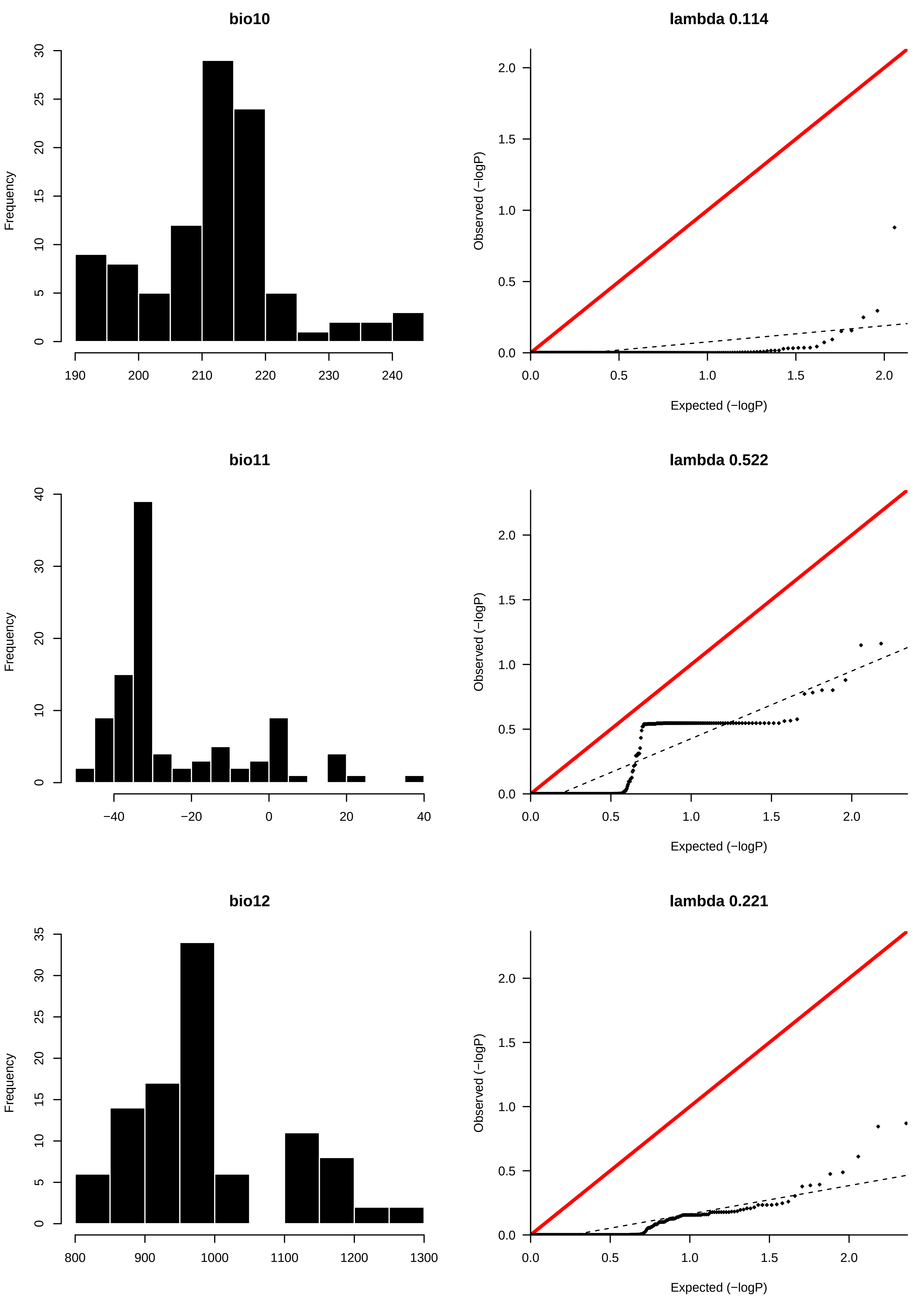
bio10
Frequency
lambda 0.114
Observed (−logP)
Expected (−logP)
bio11
Frequency
lambda 0.522
Observed (−logP)
Expected (−logP)
bio12
Frequency
lambda 0.221
Observed (−logP)
Expected (−logP)

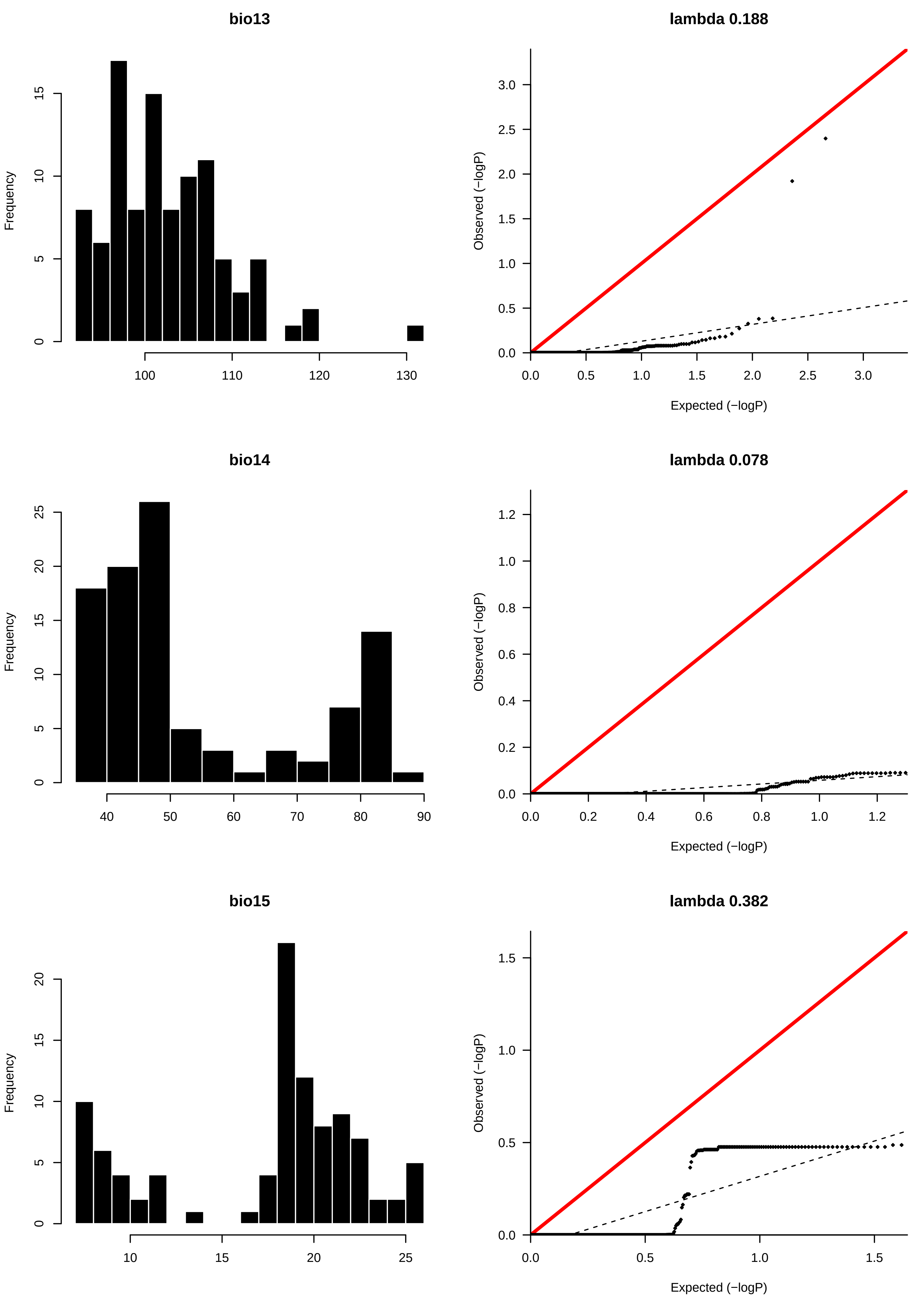
bio13
Frequency
100
110
120
130
lambda 0.188
Observed (−logP)
Expected (−logP)
bio14
Frequency
40
50
60
70
80
90
lambda 0.078
Observed (−logP)
Expected (−logP)
bio15
Frequency
10
15
20
25
lambda 0.382
Observed (−logP)
Expected (−logP)

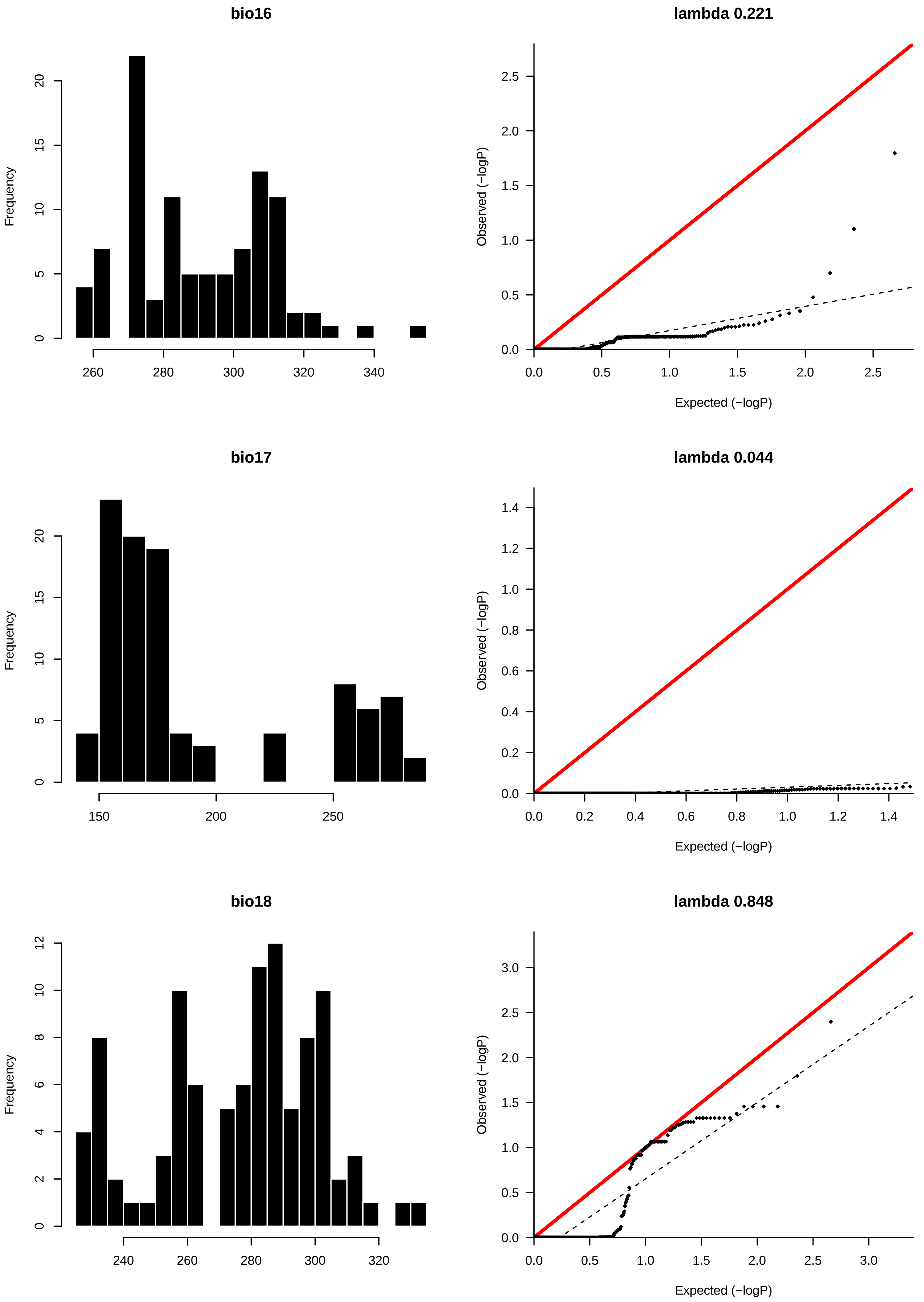
bio16
Frequency
0
5
10
15
20
260
280
300
320
340
lambda 0.221
Observed (−logP)
Expected (−logP)
0.0
0.5
1.0
1.5
2.0
2.5
bio17
Frequency
0
5
10
15
20
150
200
250
lambda 0.044
Observed (−logP)
Expected (−logP)
0.0
0.2
0.4
0.6
0.8
1.0
1.2
1.4
bio18
Frequency
0
2
4
6
8
10
12
240
260
280
300
320
lambda 0.848
Observed (−logP)
Expected (−logP)
0.0
0.5
1.0
1.5
2.0
2.5
3.0

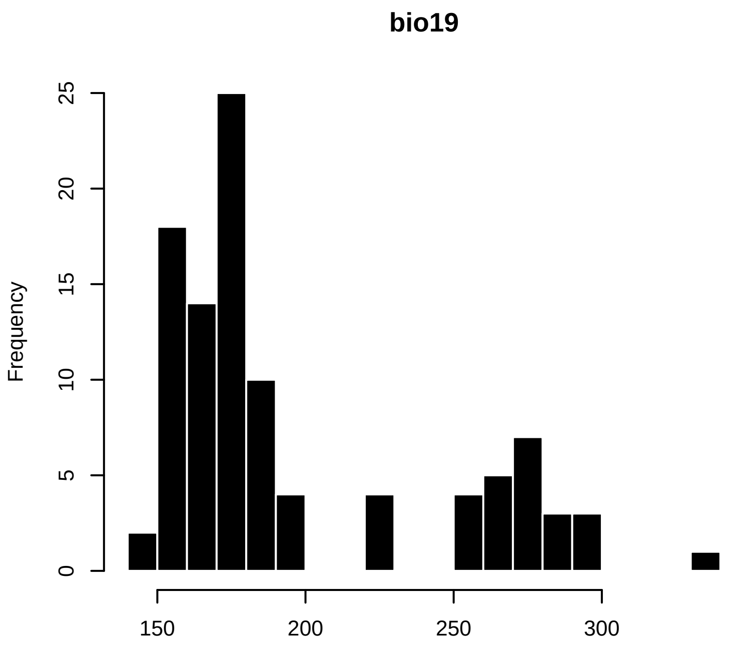
bio19
Frequency
0
5
10
15
20
25
150
200
250
300

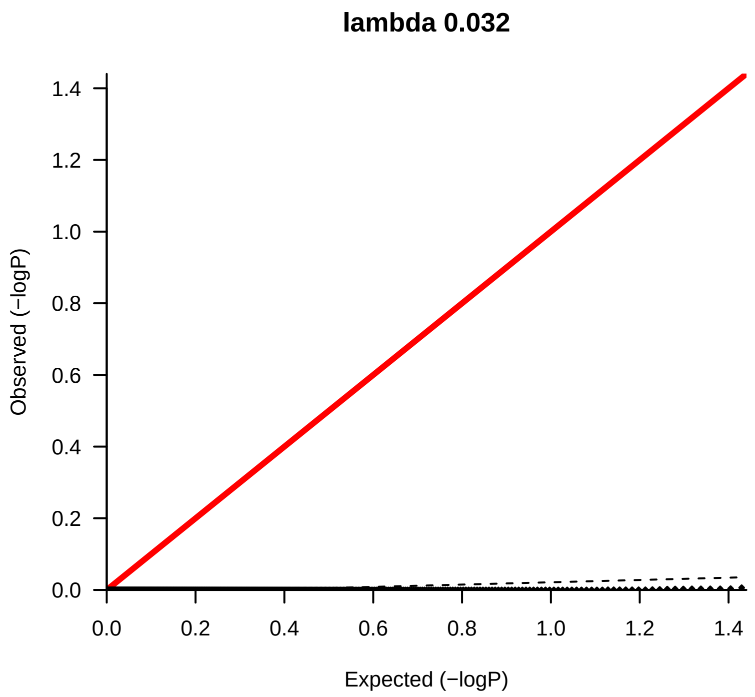
lambda 0.032
Observed (−logP)
Expected (−logP)
0.0
0.2
0.4
0.6
0.8
1.0
1.2
1.4
