# Supplementary figures and images for: The rate and potential relevance of new mutations in a colonizing plant lineage
Source: PLoS Genet. 2018 Feb 12;14(2):e1007155. doi: 10.1371/journal.pgen.1007155 (PMC5825158; doi:10.1371/journal.pgen.1007155)

**A**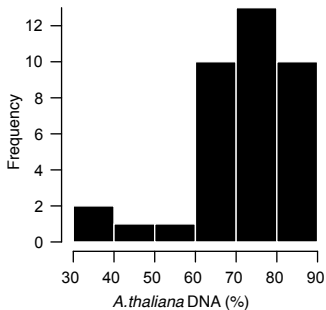**B**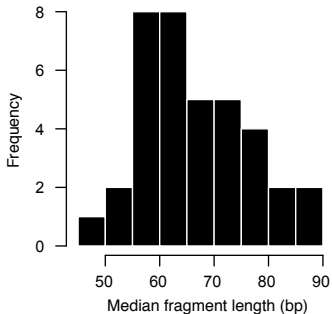**C**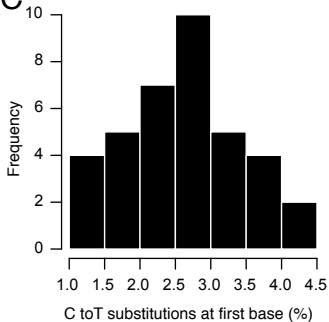**D**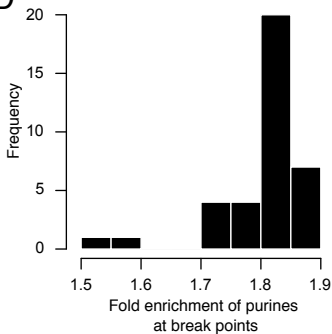

Supplement: S1 Fig — (A) Fraction of A. thaliana DNA in sample. (B) Median length of merged reads. (C) Fraction of cytosine to thymine (C-to-T) substitutions at first base (5’ end). (D) Relative enrichment of purines (adenine and guanine) at 5’ end breaking points. Position -1 is compared with position -5 (negative numbers indicate genomic context before upstream reads’ 5’ end). (PDF) [file pgen.1007155.s008.pdf]

A

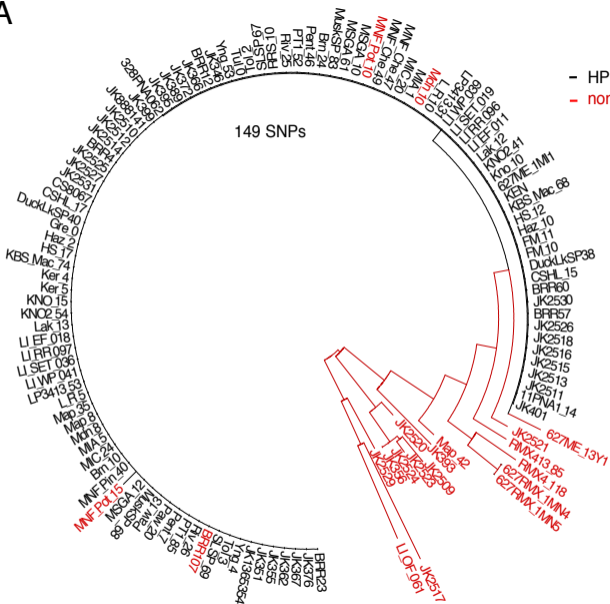

B

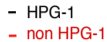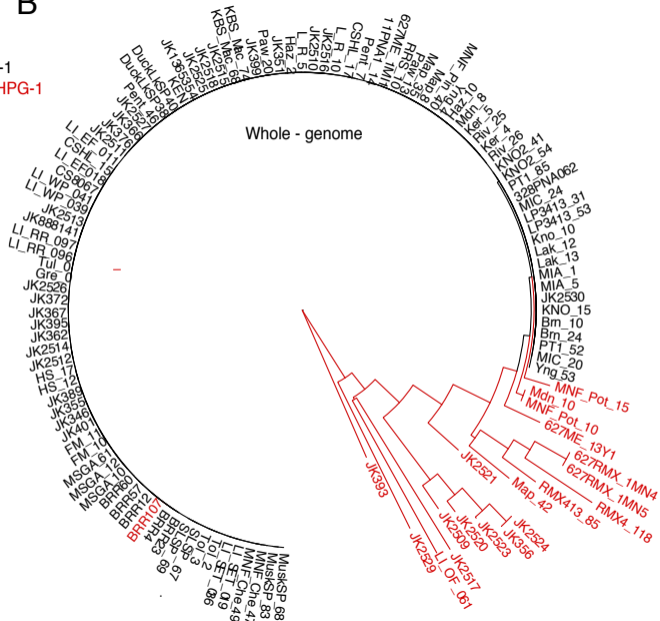

Supplement: S2 Fig — (A) Neighbor-joining tree built using Illumina-based SNP calls at the 149 genotyping markers originally used to identify HPG1 candidates. HPG1 accessions are shown in black, whereas other North American lineages are depicted in red (see explanation below for four HPG1-like accessions). (B) Neighbor-joining tree based on genome-wide SNPs. Accessions colored as in (A). Note that three accessions originally classified as HPG1 based on 149 SNPs (A) are placed outside this clade. A further accession (BRR7) within the HPG1 main branch was a recombinant removed from the analysis. (PDF) [file pgen.1007155.s009.pdf]

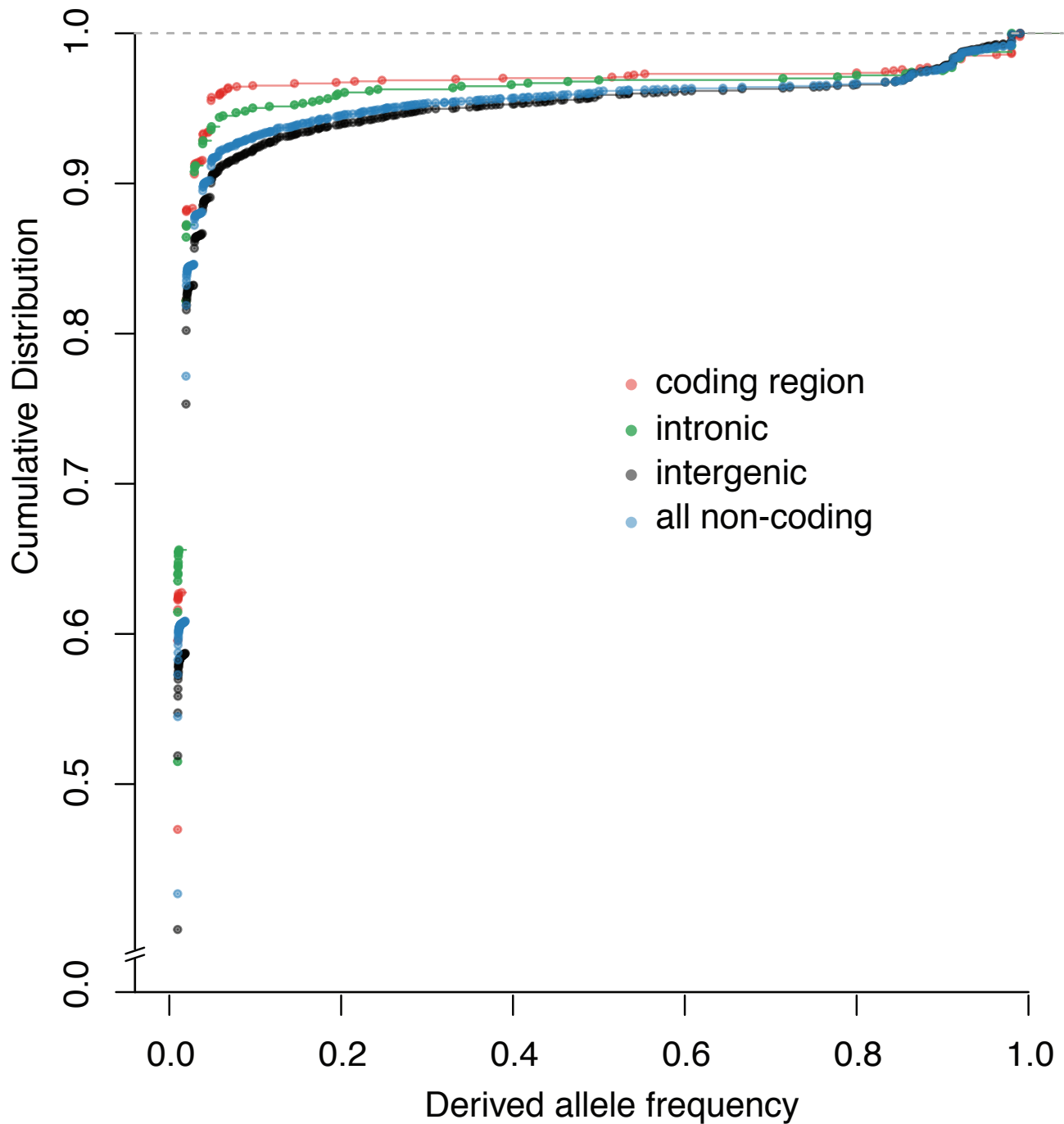

Supplement: S5 Fig — Cumulative empirical distribution, at different genomic annotations, of the unfolded Site Frequency Spectrum of SNPs oriented based on the order of appearance of alleles in the herbarium genomes. Note the steep slope at low frequency indicating large numbers of such variants. (PDF) [file pgen.1007155.s012.pdf]

A

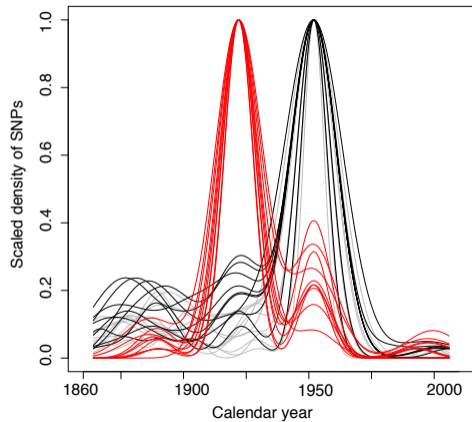

B

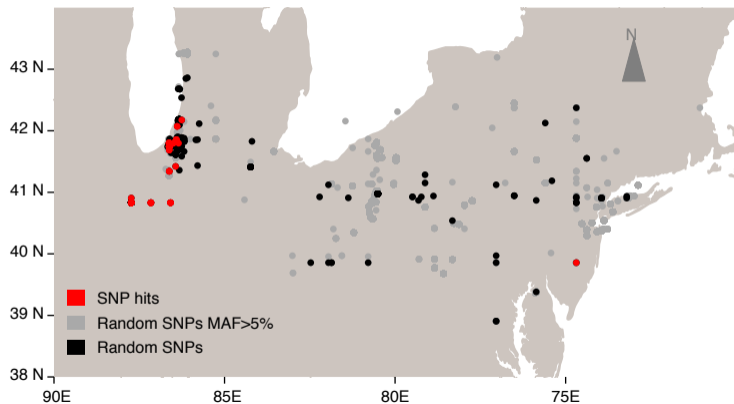

Supplement: S6 Fig — (A) Age distribution of derived SNPs with a significant trait association (the herbarium sample in which they were first recorded) (red), compared with genome-wide SNPs with at least 5% minor allele frequency (grey), or without frequency cutoff (black). (B) Spatial centroid of all samples carrying a derived allele. Since it is an average location, centroids can be in a body of water. Ten random draws of 50 SNPs for each category were used to produce the density lines in (A) and points in (B). (PDF) [file pgen.1007155.s013.pdf]

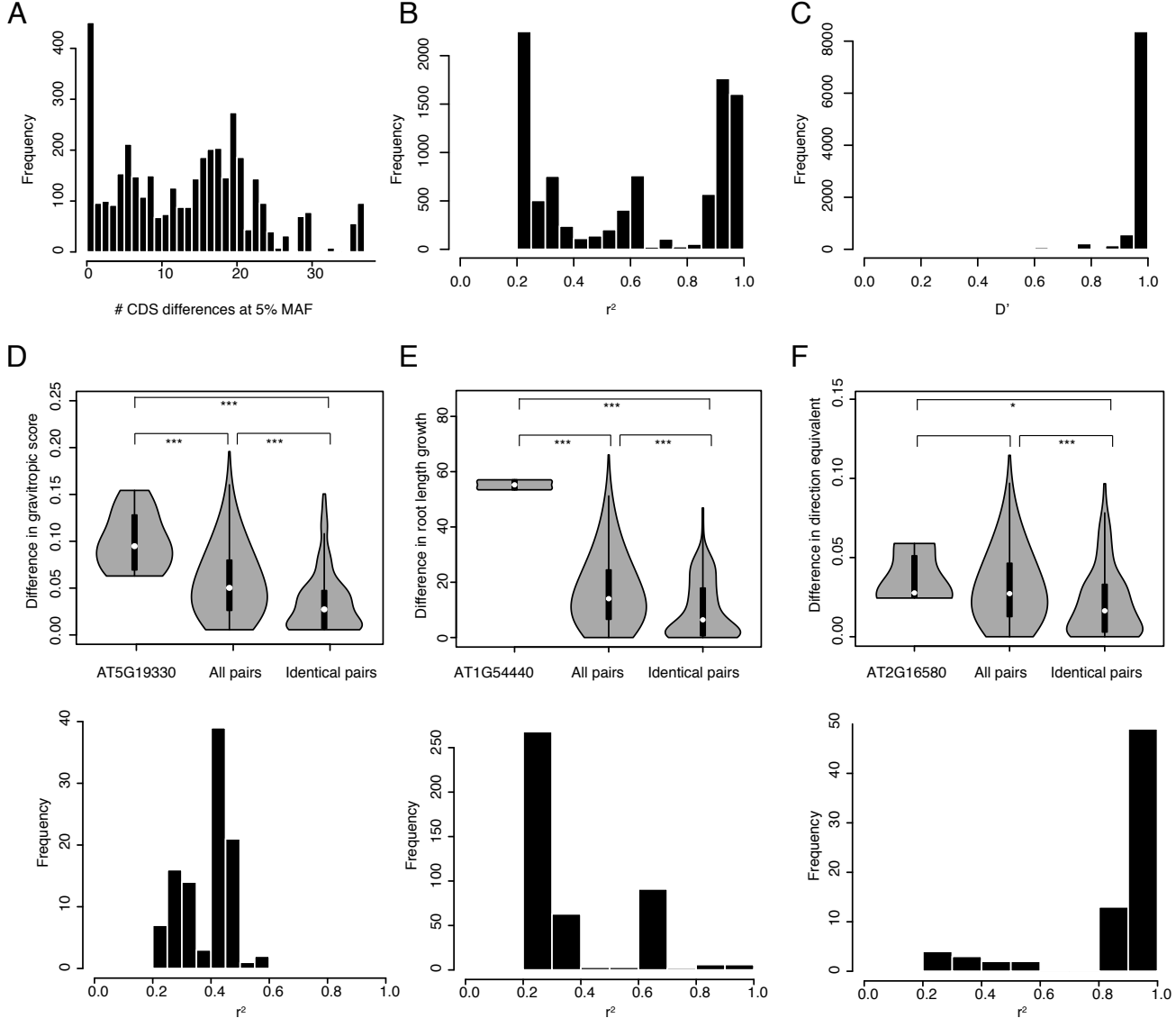

Supplement: S7 Fig — (A-F) Linkage disequilibrium between SNPs with significant trait associations. Histogram of genetic distances (A) between samples when evaluating only coding regions at 5% minimum allele frequency. Linkage disequilibrium between SNP hits measured as r2 (B) and D’ (C). Three significant SNPs were further studied to exemplify the power of association analyses with HPG1. For each, phenotypic differences between accessions that differ in the focal SNP and that are otherwise virtually genetically identical are compared both with all pairs of accessions and with pairs of accessions completely identical for coding regions. Below each violin plot is the histogram of linkage disequilibrium of the focal SNP with all other SNP hits. The three focal SNPs evaluated are located in AT5G19330 (D), AT1G54440 (E) and AT2G16580 (F). (PDF) [file pgen.1007155.s014.pdf]
