# Supplementary material for: The rate and potential relevance of new mutations in a colonizing plant lineage
Source: PLoS Genet. 2018 Feb 12;14(2):e1007155. doi: 10.1371/journal.pgen.1007155 (PMC5825158; doi:10.1371/journal.pgen.1007155)
Supplement: S4 Fig — (A, B) Fraction of methylation of cytosines in HPG1 pseudo-reference[7] at intergenic (A) or coding regions (B). (C, D) Fraction of methylation of cytosines in Col-0 reference genome(5) at intergenic (C) or coding regions (D). In each of the four comparisons, a grey histogram represents distribution of methylation of 1,000 random sets of invariant cytosines. Lines represent average methylation degree at those sites in HPG1 that changed from cytosine to thymine (red). We differentiate those substitutions that are shared—fixed—across all individuals (light red) or whose allele are present at an intermediate—segregating—frequency (dark red). Likewise, average methylation is shown for sites that changed from cytosine to adenine (blue) that that are fixed (light blue) or segregating (dark blue). The fact that the average methylation is higher in new substitutions than in invariant positions supports a connection between methylation and mutability of sites. (PDF) [file pgen.1007155.s011.pdf]

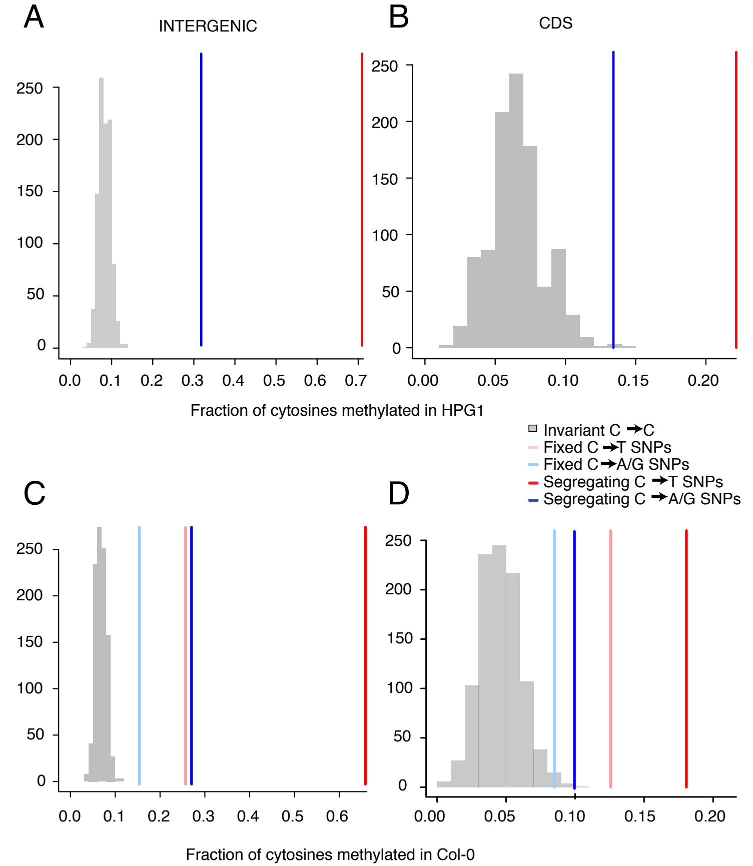

A
INTERGENIC
B
CDS
250
200
150
100
50
0
0.0 0.1 0.2 0.3 0.4 0.5 0.6 0.7
0.00 0.05 0.10 0.15 0.20
Fraction of cytosines methylated in HPG1
Invariant C →C
Fixed C →T SNPs
Fixed C→A/G SNPs
Segregating C →T SNPs
Segregating C →A/G SNPs
C
D
0.0 0.1 0.2 0.3 0.4 0.5 0.6
0.00 0.05 0.10 0.15 0.20
Fraction of cytosines methylated in Col-0
