# Supplementary material for: The rate and potential relevance of new mutations in a colonizing plant lineage
Source: PLoS Genet. 2018 Feb 12;14(2):e1007155. doi: 10.1371/journal.pgen.1007155 (PMC5825158; doi:10.1371/journal.pgen.1007155)
Supplement: S3 Fig — (A) Site frequency spectrum for all transitions and transversions. (B) Distributions of “net” pairwise genetic distances between historic and modern samples used to calculate mutation rates per genomic annotation (from quality 32_15 and complete information per site). UTRs were excluded because of the small number of SNPs. (C) Mutation rates calculated for different genomic annotations and quality thresholds (32_32, 32_15, 24_24) and missing values (NA50: maximum 50% missing data per SNP; COMPL: missing data 0%). Mean and 95% confidence intervals are shown. (PDF) [file pgen.1007155.s010.pdf]

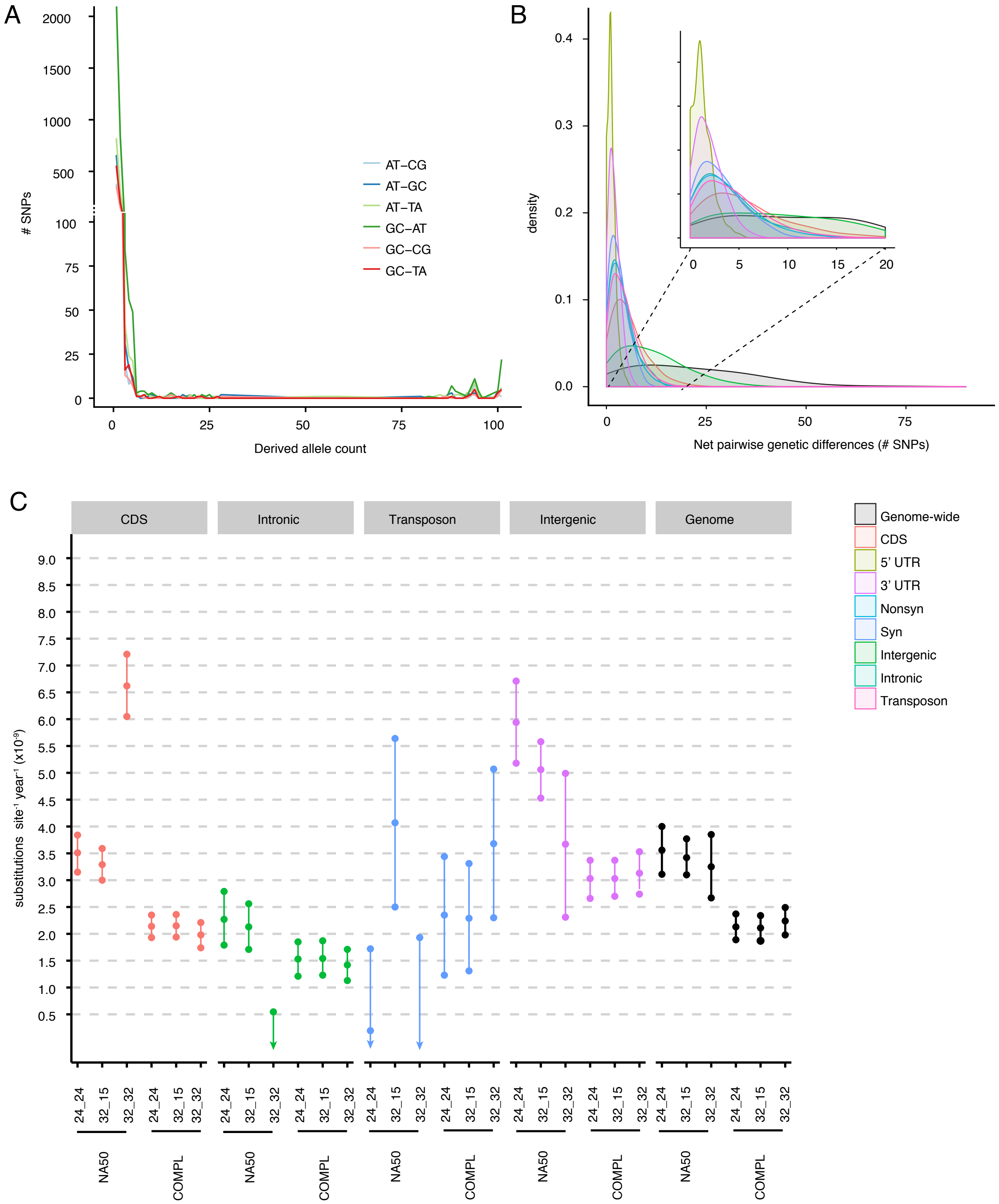

A
SNPs
Derived allele count
AT−CG
AT−GC
AT−TA
GC−AT
GC−CG
GC−TA
B
density
Net pairwise genetic differences (# SNPs)
C
CDS
Intronic
Transposon
Intergenic
Genome
substitutions site⁻¹ year⁻¹ (x10⁻⁹)
24_24
32_15
32_32
NA50
COMPL
Genome-wide
CDS
5' UTR
3' UTR
Nonsyn
Syn
Intergenic
Intronic
Transposon
